# Modulating hypertrophic scar formation by targeting endothelial transient receptor potential vanilloid-1/nuclear factor kappa-B/interleukin-6 axis to regulate angiogenesis

**DOI:** 10.1093/burnst/tkag009

**Published:** 2026-01-16

**Authors:** Hao Ma, Liuhanghang Cheng, Ruoyu Ling, Jingyi Chen, Shunuo Zhang, Shujing Lin, Liang Ding, Chengliang Deng, Yixin Zhang, Peiru Min

**Affiliations:** Department of Plastic and Reconstructive Surgery, Shanghai Ninth People’s Hospital affiliated to Shanghai Jiao Tong University School of Medicine, No. 639 Zhizaoju Road, Shanghai, 200011, China; Shanghai Institute for Plastic and Reconstructive Surgery, Shanghai Ninth People’s Hospital affiliated to Shanghai Jiao Tong University School of Medicine, No. 639 Zhizaoju Road, Shanghai, 200011, China; Department of Plastic and Reconstructive Surgery, Shanghai Ninth People’s Hospital affiliated to Shanghai Jiao Tong University School of Medicine, No. 639 Zhizaoju Road, Shanghai, 200011, China; Shanghai Institute for Plastic and Reconstructive Surgery, Shanghai Ninth People’s Hospital affiliated to Shanghai Jiao Tong University School of Medicine, No. 639 Zhizaoju Road, Shanghai, 200011, China; Department of Oral and Maxillofacial Surgery, Leiden University Medical Centre, Albinusdreef 2, 2333 ZA Leiden, the Netherlands; Department of Burn and Plastic Surgery, Southern Theater General Hospital, No. 111 Liuhua Road, Guangzhou, 510010, China; Department of Plastic and Reconstructive Surgery, Shanghai Ninth People’s Hospital affiliated to Shanghai Jiao Tong University School of Medicine, No. 639 Zhizaoju Road, Shanghai, 200011, China; Shanghai Institute for Plastic and Reconstructive Surgery, Shanghai Ninth People’s Hospital affiliated to Shanghai Jiao Tong University School of Medicine, No. 639 Zhizaoju Road, Shanghai, 200011, China; Department of Plastic and Reconstructive Surgery, Shanghai Ninth People’s Hospital affiliated to Shanghai Jiao Tong University School of Medicine, No. 639 Zhizaoju Road, Shanghai, 200011, China; School of Electronic Information and Electrical Engineering, Shanghai Jiao Tong University, 800 Dongchuan Road, Shanghai, 200240, China; State Key Laboratory for Chemistry and Molecular Engineering of Medical Resources, School of Chemistry and Pharmaceutical Sciences, Guangxi Normal University, 15 Yucai Road, Guilin, 541004, China; Department of Burns and Plastic Surgery, Affiliated Hospital of Zunyi Medical University, 149 Dalian Road, Zunyi, 563003, China; Department of Plastic and Reconstructive Surgery, Shanghai Ninth People’s Hospital affiliated to Shanghai Jiao Tong University School of Medicine, No. 639 Zhizaoju Road, Shanghai, 200011, China; Department of Plastic and Reconstructive Surgery, Shanghai Ninth People’s Hospital affiliated to Shanghai Jiao Tong University School of Medicine, No. 639 Zhizaoju Road, Shanghai, 200011, China

**Keywords:** Hypertrophic scar, Transient receptor potential vanilloid-1, Angiogenesis, Nuclear factor kappa-B, Interleukin-6

## Abstract

**Background:**

Noxious lifestyle factors including spicy diets and hot baths may lead to scar formation and recurrence. These phenomena are related to the activation of the transient receptor potential vanilloid-1 (TRPV1) cation channel. Our previous study revealed significant upregulation of TRPV1 expression in the dermis of hypertrophic scar (HS), while the exact underlying mechanism of TRPV1 activation in HS remains ill-defined. This study aims to clarify the contribution of TRPV1 activation to HS pathogenesis, particularly in relation to aberrant angiogenesis.

**Methods:**

First, this study employs single-cell RNA sequencing technology to analyze the association between vascular endothelial cells and the development of HS. Complementarily, bioinformatics analysis combined with histological validation is utilized to investigate the relationship between TRPV1 channels and aberrant angiogenesis within HS formation. Furthermore, the correlation between TRPV1 activation and HS phenotypes is rigorously validated at the *in vivo* level. In parallel, *in vitro* experiments are conducted to elucidate the impact of TRPV1 channel activation on the biological behaviors and functions of vascular endothelial cells. Subsequently, key downstream signaling pathways of TRPV1 are screened, and their molecular mechanisms in regulating vascular endothelial cell-mediated angiogenesis are systematically verified. Finally, a comprehensive analysis is performed to establish the clinical relevance of the TRPV1/nuclear factor kappa-B (NF-κB)/interleukin-6 (IL-6) axis with vascularization severity and adverse prognostic outcomes in hypertrophic scarring.

**Results:**

Single-cell RNA sequencing revealed significant cellular heterogeneity in vascular endothelial cells between normal skin and HS, indicating activated angiogenesis and substantial vascular endothelial cell alterations during HS development. Bulk RNA-seq and clinical analyses further confirmed this angiogenesis activation, demonstrating a close association with TRPV1 channel activation. *In vivo* studies established that capsaicin (CAP)-induced TRPV1 activation exacerbated HS progression through enhanced angiogenesis, whereas TRPV1 ablation or local inhibition markedly attenuated this effect. *In vitro* experiments demonstrated that TRPV1 activation regulated angiogenesis by promoting pro-angiogenic phenotypes. Transcriptomic analysis and functional validation identified the IL-6/signal transducer and activator of transcription 3 pathway as a downstream NF-κB-dependent pro-angiogenic axis mediated by TRPV1 in HS vascular endothelial cells. Critically, dermal overexpression of the TRPV1/NF-κB/IL-6 axis in HS patients correlated strongly with both disease severity and recurrence.

**Conclusions:**

Here, we show that the development of HS is strongly correlated with endothelial angiogenic activity. TRPV1 activation by CAP enhances proangiogenic processes including endothelial proliferation, migration, and tubule formation, while reducing apoptosis through the TRPV1/NF-κB/IL-6 axis. In a rabbit ear HS model, stimulation of TRPV1 contributes to the formation of HS via the TRPV1/NF-κB/IL-6 axis, whereas pharmacological ablation of TRPV1 significantly reversed these phenotypes. These findings shed light on the underlying molecular mechanisms and provide a potential therapeutic target for HS.

## Graphical Abstract

**Graphical Abstract:**
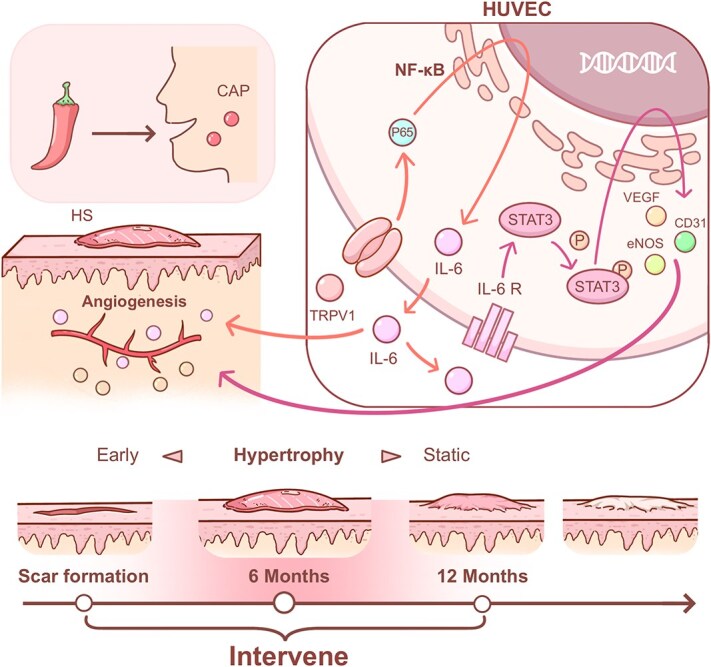

## Highlights

- This study reveals distinct differences in the phenotypic profiles of vascular endothelial cells between NS and HS.
- CAP exposure modulates vascular endothelial cell function via the TRPV1 signaling pathway, ultimately exacerbating the pathogenesis of hypertrophic scarring.
- We provide a mechanistic framework demonstrating that TRPV1 channel activation drives HS development by promoting NF-κB/IL-6 axis-mediated angiogenesis.
- The TRPV1 channel and its downstream effectors NF-κB and IL-6 are identified as potential novel diagnostic biomarkers, prognostic indicators, and therapeutic targets for hypertrophic scarring.

## Background

Hypertrophic scarring usually occurs secondary to skin wounds including trauma, burns and surgery, imposing a huge burden on patient health and economy well-being. Although traditionally characterized by abnormal fibroblasts proliferation and extracellular matrix (ECM) deposition, hypertrophic scar (HS) formation has been verified to highly correlate with increased vascularity and dysfunctional endothelial heterogeneity [1, 2]. Rapid vascularization is beneficial for nutrient and cytokine transportation at the early stage of wound healing, whereas uncontrolled vessel growth and impaired vessel regression lead to excessive inflammatory responses and result in dysfunction and scarring of the skin architecture during the proliferation and remodeling phase [1]. Endothelial cells are activated and release various vascular secretory factors, including vascular endothelial growth factor (VEGF), platelet-derived growth factor, and endothelial nitric oxide synthase (eNOS) [3]. These factors may further facilitate cross-talk with surrounding fibroblasts, macrophages, and epithelial cells, thus accelerating scar formation [4]. As a result, the appropriate limitation of angiogenesis is considered a key factor in modulating HS disease [1].

As scar formation is a multifaceted, multifactorial, and tightly controlled process, multiple risk factors including ethnicity, sex, age, and location may contribute to disease progression. For example, Asians and Africans are highly prone to HS, with estrogen in females stimulating HS progression, especially during pregnancy [5, 6]. Moreover, although HS can develop at any age and anatomical site, the incidence peaks between 10 and 30 years of age, with scars frequently being stretched by daily body movements [7]. The HS typically emerges within 1-month postinjury, undergoes progressive growth within 12 months, and subsequently enters a stabilization phase [8]. Notably, clinical evidence has indicated that lifestyle factors such as consumption of spicy foods and prolonged hot baths can aggravate scar hypertrophy and recurrence [7]. Transient receptor potential vanilloid-1 (TRPV1) is a polymodal nonselective cation channel, whose selective agonist is named capsaicin (CAP) [9]. The cation channel is broadly localized to human organs including the central nervous system, kidney, pancreas, lung and liver, where they play critical roles in response to external environmental stimuli and affect the development of different types of tumors [10]. In our previous work, we reported a widespread overexpression of TRPV1 in activated pathological scar sites, along with the downstream peri-vascular neuropeptide Substance P (SP) [11]. Although SP release was also observed in acute burn injury and may induce local vasodilatation and plasma extravasation, the exact mechanism of TRPV1 overexpression and local angiogenesis stimulation in HS stands deleterious [12]. Interestingly, TRPV1 regulates vascular remodeling and promotes angiogenesis through different pathways involving nitric oxide and calcitonin gene-related peptide in cardiac and renal disease, and in tympanic membrane repair, it promotes macrophage-associated angiogenesis via monocyte recruitment [13, 14]. It has been demonstrated to be broadly expressed in skin non-neuronal tissue including blood vessels, and to release pro-inflammatory agents, followed by sensitization of channels and triggering of pain signals [15]. Moreover, the TRPV1 channel can be activated not only by CAP but also by environmental inputs including heat (more than 43°C), pH, and Ultraviolet (UV) light, which are highly correlated with the clinical features of pathological scars [16]. As TRPV1 deficiency resulted in retarded re-epithelialization and delayed cutaneous wound healing, the potential contribution of TRPV1 to human HS remains enigmatic [12, 17].

Opening of the TRPV1 channel in the plasma membrane may cause the entry of extracellular Ca^2+^ down the electrochemical gradient, which results in an increase in the cytosolic Ca^2+^ concentration. Thereafter, the Ca^2+^-sensitive decoders activate signaling transcription factors including nuclear factor kappa-B (NF-κB), protein kinase A (PKA), and mitogen-activated protein kinase [18]. For example, activation of TRPV1 has been shown to induce the phosphorylation of eNOS in a PKA-dependent manner thus mediating the function of endothelial cells [19]. However, the exact underlying molecular mechanism mediated by TRPV1 in HS is still ill-defined.

Interleukin-6 (IL-6) upregulation-mediated inflammation is considered a key player in the development of keloids [20]. Here, our study demonstrated that activation of TRPV1 enhances angiogenesis and HS formation mediated by the TRPV1/NF-κB/IL-6 axis in endothelial cells. Clinical specimens and *in vivo* experimental results revealed that the activation of TRPV1 could accelerate angiogenesis and HS formation. *In vitro* experimental evidence illustrated an increase in pro-angiogenic function in terms of endothelial proliferation, migration, and tubule formation with a decrease in apoptosis. The bioinformatics results suggest a connection between the function of endothelial cells mediated by TRPV1/NF-κB/IL-6 axis and this hypothesis was verified through *in vivo* and *in vitro* experiments. Thus, endothelial TRPV1 is a potential therapeutic target in the management of HS disease.

## Methods

### Patients and samples

Normal skin (NS) samples and HS samples were all obtained from patients treated in the Department of Plastic and Reconstructive Surgery, Shanghai Ninth People’s Hospital. This study received approval from the Independent Ethics Committee of Shanghai Ninth People’s Hospital, which is affiliated with Shanghai Jiao Tong University School of Medicine, and informed consent was secured from all patients (SH9H-2024-T319-2). Only newly developed and proliferative HS tissue samples from patients who underwent elective scar resection surgery were included in this study.

We collected three NS samples and three HS samples for single-cell sequencing analysis (Table S1, see online supplementary material). The tissues were transferred into the culture dish and cut into 0.5 mm^2^ pieces; then the tissues were washed with 1 × phosphate buffer saline (PBS) to remove as many non-target tissues as possible, such as blood stains and fatty layers. In addition, we also collected three NS samples and three HS samples for bulk RNA sequencing analysis (Table S2, see online supplementary material). Meanwhile, two additional bulk RNA sequencing datasets used in this study of NS and HS samples were collected from the Gene Expression Omnibus with accession numbers GSE181540 and GSE178411.

Besides, we followed and monitored 20 HS patients who underwent scar resection surgery for a duration of 2 years (Table S3, see online supplementary material). Pearson’s correlation analysis and the receiver operating characteristic (ROC) curve were performed to detect the clinical characteristics. For correlation analyses examining linear relationships between clinical parameters, paired measurements were organized in Column tables where Pearson correlation coefficients with two-tailed *P*-values and 95% confidence intervals were computed via the XY analysis module after verifying bivariate normal distribution using D’Agostino-Pearson omnibus tests (α = 0.05). ROC curves evaluating diagnostic performance were generated through the Column analysis platform, wherein patient and control data were segregated, enabling calculation of area under the curve (AUC) with 95% confidence intervals by DeLong’s method, sensitivity/specificity metrics, and optimal cut-off values determined by maximizing Youden’s index (J = sensitivity + specificity−1).

### Single-cell RNA sequencing and analysis

#### Tissue dissociation and preparation of single-cell suspensions

Tissues were dissociated into single cells in a dissociation solution in a 37°C water bath with shaking for 20 min at 100 rpm. Digestion was terminated with 1 × PBS containing 10% fetal bovine serum, then pipetting 5–10 times with a Pasteur pipette. The resulting cell suspension was filtered and centrifuged at 300 *g* for 5 min at 4°C. The cell pellet was resuspended in 100 μl 1 × PBS (0.04% bovine serum albumin (BSA)) and added with 1 ml 1 × red blood cell lysis buffer and incubated at room temperature or on ice for 2–10 min to lyse the remaining red blood cells. After incubation, the suspension was centrifuged at 300 *g* for 5 min at room temperature. The suspension was resuspended in 100 μl of Dead Cell Removal MicroBeads (MACS 130-090-101) and removed dead cells using the Miltenyi® Dead Cell Removal Kit (MACS 130-090-101) with Large Scale (LS) columns under a magnetic field strength >12 000 Gauss, repeating the separation twice to achieve >95% dead cell clearance. Then the suspension was resuspended in 1 × PBS (0.04% BSA) and centrifuged at 300 *g* for 3 min at 4°C(repeat twice). The cell pellet was resuspended in 50 μl of 1 × PBS (0.04% BSA). The overall cell viability was confirmed by trypan blue exclusion and was required to be above 85%; single-cell suspensions were then counted using a hemocytometer, and the concentration was adjusted to 700–1200 cells/μL.

#### Chromium 10x genomics library and sequencing

Single-cell suspensions were loaded onto the 10x Chromium according to the manufacturer’s instructions of 10x Genomics Chromium Single-cell 3′ kit (V3). The following cDNA amplification and library construction steps were performed according to the standard protocol. Libraries were sequenced on an Illumina NovaSeq 6000 sequencing system (paired-end multiplexing run,150 bp) by LC-Bio Technology Co. Ltd (Hangzhou, China).

#### Bioinformatics analysis

Our own sequencing results were demultiplexed and converted to FASTQ format using Illumina bcl2fastq software (version 2.20). Sample demultiplexing, barcode processing, and single-cell 3′ gene counting were performed using the Cell Ranger pipeline (https://support.10xgenomics.com/single-cell-gene-expression/software/overview/welcome). The resulting data were aligned to the Ensembl genome GRCh38. A total of 67 827 single cells captured from three NS donors and three HS patients were processed using the 10X Genomics Chromium Single Cell 3′ Solution.

To expand our statistical power and ensure biological consistency, we integrated our dataset with a publicly available single-cell RNA-seq (scRNA-seq) dataset (GSE156326). We applied a standardized quality control workflow to both datasets: all genes expressed in fewer than three cells were excluded; cells with fewer than 500 or more than 3000 genes per cell were removed; cells with Unique Molecular Identifier (UMI) counts below 500 were excluded; and cells with mitochondrial-derived gene expression exceeding 25% were filtered out.

Overall, we retained 79, 281 cells (Our own database: 56854 cells, GSE156326: 22427 cells) and utilized the Seurat v3.1.1 package to perform log-normalization followed by principal component analysis (PCA).

Based on the analysis results (determining the integration anchor points), we implemented a robust integration strategy utilizing the Harmony algorithm (v1.2.3) to harmonize transcriptomic profiles across platforms and time points. Specifically, after conducting PCA on the quality-controlled data to identify the major sources of biological variation, we determined the integration anchor points via reciprocal PCA. Subsequently, we utilized the Integrate Layers function with the Harmony method to effectively mitigate batch effects by identifying shared latent factors while preserving biological differences.

Uniform manifold approximation and projection (UMAP) and clustering were subsequently applied to the unified dataset for projecting the cells onto 2D space. The steps include: (i) using the LogNormalize method of the “Normalization” function of the Seurat software to calculate the gene expression values; (ii) PCA was performed using the normalized expression value, and among all the PCs, the top 10 PCs were used for clustering and t-distributed stochastic neighbor embedding (t-SNE) analysis; (iii) identifying clusters using a weighted shared nearest neighbor graph-based clustering method. Marker genes for each cluster were identified with the Wilcoxon rank-sum test (default parameters is “bimod”: Likelihood-ratio test) with default parameters via the FindAllMarkers function in Seurat. This selects marker genes that are expressed in more than 10% of the cells in a cluster and have an average log(Fold-Change) greater than 0.25 (default parameters: 0.26).

Gene ontology (GO) enrichment and Kyoto Encyclopedia of Genes and Genomes (KEGG) pathways of differentially expressed genes (DEGs) at *P* < 0.05 were analyzed using a R package clusterProfiler v3.12.0 [21]. Bubble plots were generated using ggplot2 (v3.4.0) to visualize the top enriched GO terms, while KEGG pathways were reconstructed using pathview (v1.24.0) to highlight the involvement of specific genes in signaling processes. Gene Set Enrichment Analysis (GSEA) was also conducted using the Molecular Signatures Database (http://gsea-msigdb.org), where normalized enrichment scores (NES) were acquired using gene set permutations performed 1,000 times, and a cutoff *P*-value of 0.05 and false discovery rate (FDR) < 0.25 were used to filter the significant enrichment results.

### Bulk RNA sequencing and analysis

#### Sample preparation and RNA sequencing

After careful removal of the epidermis and subcutaneous adipose tissue with a scalpel, three NS samples and three HS samples were stored in a storage reagent (RNAlater, Invitrogen, CA) at −20°C for bulk RNA sequencing analysis. We also collected human umbilical vein endothelial cells (HUVECs) cultured with or without CAP for bulk RNA sequencing analysis.

Total RNA was extracted from both tissue and cell samples using TRIzol reagent (Thermo Fisher, 15596018), following the manufacturer’s procedure. Total RNA quantity and purity were analyzed using a Bioanalyzer 2100 and the RNA 6000 Nano LabChip Kit (Agilent, CA, USA, 5067-1511), and high-quality RNA samples with an RNA integrity number (RIN) > 7.0 were used to construct sequencing libraries. Prior to library construction, rRNA was depleted using the Ribo-Zero™ rRNA Removal Kit (EPICENTRE, WI) to ensure accurate quantification of mRNA transcripts. We performed the 2 × 150 bp paired-end sequencing (PE150) on an Illumina NovaSeq™ 6000 (LC-Bio Technology CO., Ltd, Hangzhou, China) following the vendor’s recommended protocol.

Raw sequencing data were quality-trimmed using Fastp (v0.20.0) to remove poly-N reads and low-quality bases (Q < 20), and adapter sequences were trimmed using Trimmomatic (v0.39) to ensure high-quality input for downstream analysis.

#### Bioinformatics analysis

DEGs were identified using DESeq2 R package with default settings, including median-of-ratios normalization and variance stabilization to account for technical variability [22]. Changes in gene expression with a *P*-value < 0.05 and |log2(Fold-Change)| >1 or Fold-Change >1.5 were considered statistically significant. GO enrichment and KEGG pathways of DEGs at *P* < 0.05 were analyzed using a R package clusterProfiler v3.12.0 [21]. GSEA analysis was also conducted using the Molecular Signature Database (http://gsea-msigdb.org), where Normalized enrichment scores were acquired using gene set permutations performed 1000 times, and a cutoff *P*-value of 0.05 and FDR < 0.25 were used to filter the significant enrichment results. Venn diagrams showing the overlap of different gene sets were generated by Venny v2.1 (https://bioinfogp.cnb.csic.es/tools/venny). The protein–protein interaction (PPI) network and the enrichment interaction network were constructed for genes in the GO terms of DEGs. To explore downstream functional implications, the genes associated with significant GO term were extracted and used to construct a PPI network using the STRING database (https://string-db.org/) with a confidence score threshold of >0.4. The PPI network was visualized and refined in Cytoscape 3.8.2, and the enrichment interaction network was generated by integrating the PPI network with GO term annotations.

### Animal models

The rabbit ear HS model was established according to the previously described method with approval from the Animal Experimentation Ethics Committee of the School of Medicine, Shanghai Jiao Tong University (SH9H-2023-A513-SB) [23]. Briefly, adult New Zealand White rabbits (2.0–2.5 kg, Si-Lai-Ke, Shanghai, China) received intravenous anesthesia with pentobarbital sodium through their ear veins (30 mg/kg). Four wounds (10 mm in diameter) extending to the bare cartilage were created on the ventral side of each ear, with the overlying perichondrium excised. Following the procedure, the rabbits were placed in individual cages, permitted free movement, and provided with a standard diet. Sterile gauze was applied to cover the wounds for a duration of 1 day.

### *In vivo* capsaicin and resiniferatoxin treatment

The therapeutic effects of the TRPV1 channel agonists CAP (Abcam, ab141000) and resiniferatoxin (RTX) (Alomone Labs, R-400) were evaluated in a rabbit ear HS model. We injected 50, 100, 200 mg/kg CAP subcutaneously into adult New Zealand White rabbits (1 week before and after the surgery) in the dorsal thoracic region. Systemic ablation of the TRPV1 channel was induced by treatment with RTX, as previously described [24, 25]. Thus, we injected three escalating doses (30, 70, and 100 μg/kg) subcutaneously into adult New Zealand White rabbits in the dorsal thoracic region on consecutive days. Rabbits were allowed to rest for 4 weeks before surgery. Additionally, the control New Zealand White rabbits were treated with PBS solution.

### *In vivo* capsazepine treatment

The therapeutic effects of the TRPV1 blocker capsazepine (CPZ) (MCE, HY-15640) were evaluated in a rabbit ear HS model. The solubility of CPZ ranges from 7.61 to 13.27 mM (2.87 to 5 mg/ml) and there is not yet a reference article about the experimental approaches of CPZ in the field of HS disease research. For our experimental investigation, four concentrations (0, 40 μg/ml, 400 μg/ml, and 4 mg/ml) were selected to assess its efficacy in treating HS. To activate the TRPV1 channel, 100 mg/kg of CAP was injected subcutaneously into the dorsal thoracic region of adult New Zealand White rabbits 1 week before and after surgery. Subsequently, 50 μl of CPZ was locally injected at the specified concentrations into rabbit ears at 2 and 3 weeks after surgery.

### *In vivo* axitinib treatment

The therapeutic effects of the antiangiogenic drug axitinib (VEGFR inhibitor, Selleck Chemicals, AG-013736) were evaluated in a rabbit ear HS model. Based on previous studies of axitinib-mediated scar inhibition after HS formation, 1.25 mg/ml of axitinib was locally injected into the scar lesions at 2 and 3 weeks after surgery [26].

### Histological analysis

Four weeks after the establishment of HS models, all remaining rabbits were sacrificed, and the tissues of formed HS were obtained for histological analysis. The tissues were fixed in 4% paraformaldehyde for tissue fixation. After being dehydrated with a series of graded ethanol, the tissues were embedded in paraffin and cut into 5-μm-thick longitudinal sections. Then, the sections were stained with H&E staining, Masson Trichrome staining, and Sirius Red staining. The image was taken by a microscope (Nikon, Japan), while the scar evaluation index (SEI), percentage of collagen, and type III/I collagen ratio were analyzed by ImageJ.

### Immunohistochemistry

The expression of α-smooth muscle actin (α-SMA) in rabbit ear HS tissues was examined using a commercially validated anti-α-SMA antibody (Invitrogen, MA1-06110) by a standard immunohistochemistry protocol. Following antigen retrieval in citrate buffer (pH 6.0, 95°C, 15 min) and blocking of endogenous peroxidase with 3% H₂O₂, dewaxed sections were incubated with primary anti-α-SMA antibody (Invitrogen, MA1-06110) at a dilution of 1:250 for 1 h at room temperature after non-specific binding inhibition with 5% BSA, followed by incubation with goat anti-mouse immunoglobulin G-horseradish peroxidase (IgG-HRP) secondary antibody (Abcam, ab6789) for 30 min. Images were taken by a microscope (Nikon, Japan). Quantification of α-SMA expression area was performed by calculating the percentage of diaminobenzidine (DAB)-positive area relative to total tissue area using ImageJ (threshold-based segmentation). The analysis of CD31 and vascular endothelial growth factor A (VEGFA) was performed in the same way using an anti-CD31 antibody (Abcam, ab182981, a dilution of 1:200) and an anti-VEGFA antibody (Abcam, ab1316, a dilution of 1:200), with identical quantification of positive area ratio.

The expression of TRPV1 in human HS and NS tissues was examined using a commercially validated anti-TRPV1 antibody (Alomone, ACC030) by a standard immunohistochemistry protocol. Briefly, dewaxed sections underwent uniform antigen retrieval and blocking procedures before incubation with primary antibodies (TRPV1 at 1:200) and species-matched HRP secondaries. Images were taken by a microscope (Nikon, Japan). TRPV1-positive area was quantified as the percentage of stained area per total tissue section. The analysis of NF-κB and IL-6 was performed in the same way using an anti-NF-κB p65 antibody (CST, 8242, a dilution of 1:200) and an anti-IL-6 antibody (CST, 12912, a dilution of 1:200), using identical area-based quantification methods.

### Immunofluorescence

Co-immunofluorescence (IF) for TRPV1 (Alomone, ACC030) and CD31 (Abcam, ab9498) was performed on collected rabbit HS samples. After being deparaffinized and rehydrated, scar sections were subjected to ethylenediaminetetraacetic acid (EDTA) antigen retrieval solution (Solarbio, China), and the samples were blocked by H_2_O_2_ and goat serum (Beyotime, China). Then, the samples were co-incubated with primary rabbit antibody against TRPV1 (Alomone, ACC030) and primary mouse antibody against CD31 (Abcam, ab9498) overnight at 4°C. After three washes with PBS, Alexa Fluor 488-conjugated goat anti-rabbit secondary antibodies (Abcam, ab150077, 1:1000) and Alexa Fluor 594-conjugated goat anti-mouse secondary antibodies (Invitrogen, A-11005, 1:1000) were incubated for 45 min in the dark at 37°C at room temperature for 1 h. Blue-fluorescent 4′,6-diamidino-2-phenylindole (DAPI) dye (1 μg/mL) was added to the plates for 10 min to stain cell nuclei before the end of the incubation. The expressions of TRPV1(green emission signal) and CD31 (red emission signal) were then observed using an upright IF microscope (Zeiss, Germany). Standard IF of NF-κB p65 for rabbit HS samples was performed using an anti-NF-κB p65 primary antibody (CST, 8242) and an Alexa Fluor 488-conjugated goat anti-rabbit secondary antibody (Abcam, ab150077, 1:1000).

For human sections, co-IF for TRPV1 (Alomone, ACC030) and CD31 (Abcam, ab9498), as well as for NF-κB (CST, 8242) and CD31 (Abcam, ab9498), was performed on collected NS and HS samples in the same way as described above.

HUVECs were placed on coverslips in 24-well plates and incubated in 5% CO_2_ and humidified air at 37°C. After treatment with CAP, HUVECs were fixed with 4% paraformaldehyde for 30 min and permeabilized with 0.1% Triton X-100 for 30 min at 25°C. Then, the cells were incubated overnight at 4°C with primary anti-NF-κB p65 antibody (CST, 8242, 1:100). After three washes with PBS, cells were incubated with the working solution, including Alexa Fluor 488-conjugated goat anti-rabbit secondary antibodies (Abcam, ab150077, 1:1000) at room temperature for 1 h. Blue-fluorescent DAPI dye (1 μg/mL) was added to the plates for 10 min to stain cell nuclei before the end of incubation. The stained slides were observed and captured using an inverted IF microscope (Zeiss, Germany). Fluorescence images of the stained cells were acquired using the excitation wavelengths of 488 nm (green) and 405 nm (blue).

### RNA isolation and quantitative real-time PCR

Total RNA was extracted from both tissues and cells using TRIzol reagent (Invitrogen, USA) according to the manufacturer’s protocol. Then, 1000 ng of total RNA was reverse transcribed into complementary DNA (cDNA) using the FastKing cDNA synthesis kit (TIANGEN Biotech, China). Quantitative real-time PCR (q-PCR) was performed with TB Green Premix (Takara, Japan). The mRNA expression levels of TRPV1, IL-6, CD31, VEGFA, CD34, vascular endothelial growth factor receptor (VEGFR), and eNOS in human samples, and collagen type I alpha 1 chain (COL1A1), transforming growth factor beta (TGF-β), fibronectin, α-SMA, CD31, VEGFA, CD34, VEGFR, eNOS, TRPV1, and IL-6 in rabbit ear samples were detected. Polymerase chain reaction (PCR) primers were designed based on sequences from the corresponding genes (Table S4, see online supplementary material). All data were normalized using glyceraldehyde-3-phosphate dehydrogenase (GAPDH) as the internal control by the ΔCT method. Relative expression was calculated for each gene by the 2^−ΔΔCT^ relative quantification method. Gene expression levels were normalized to the endogenous reference gene GAPDH. GAPDH was selected as the reference gene based on its widespread use and demonstrated stability in studies involving human endothelial cells and mammalian dermal tissues, and to maintain consistency with our protein expression normalization.

### Western blotting

Total proteins were extracted using radioimmunoprecipitation assay (RIPA) lysis buffer containing phosphatase and protease inhibitors (Fude Biotechnology, China). The concentration of total protein was detected with a bicinchoninic acid (BCA) Protein Assay kit (Beyotime Biotechnology, China), and equal amounts (20 μg) of protein were loaded onto sodium dodecyl sulfate–polyacrylamide gel electrophoresis (SDS-PAGE) gels to ensure consistent protein loading across all lanes. Proteins were transferred to nitrocellulose membranes (0.45 μm; Millipore, USA) and blocked with 5% non-fat milk for 1 h at room temperature. Primary antibodies were incubated overnight at 4°C under strictly controlled conditions, including the use of unified antibody batches. The following primary antibodies were used: anti-GAPDH antibody (Abcam, ab8245, 1:2000), anti-α-SMA antibody (Abcam, ab7817, 1 μg/ml), anti-col1a1 antibody (Proteintech, 66761-1-Ig; CST, 72026, 1:1000), anti-VEGFR2 antibody (Invitrogen, MA5-15157, 1:500), anti-CD31 antibody (Abcam, ab182981, 1:2000; Abcam, ab9498, 1:2000), anti-TRPV1 antibody (Abcam, ab305299, 1:2000), anti-IL-6 antibody (CST, 12912, 1:1000; R&D, MAB206-SP, 8 μg/ml), anti-signal transducer and activator of transcription 3 (STAT3) antibody (CST,4904, 1:1000), anti-Phospho-STAT3(Tyr705) antibody (CST, 9145, 1:1000), anti-NF-κB p65 antibody (CST, 8242, 1: 1000). After washing, membranes were probed with goat anti-rabbit IgG H&L secondary antibody (Abcam, ab6702, 1:5000) or goat anti-mouse IgG H&L secondary antibody (Abcam, ab6708, 1:5000). Detection was performed using the Odyssey CLx Infrared Imaging System (LI-COR Biosciences, USA). To ensure quantitative accuracy, standardized exposure times within the linear detection range were utilized, and band intensities were analyzed using ImageJ software. For all Western blot experiments, we strictly adhered to standardized protocols, including triplicate biological replicates for each condition, to validate the reproducibility of our findings. GAPDH served as the loading control to normalize protein loading.

### Cell culture

HUVECs and human foreskin fibroblast-1 (HFF-1) were obtained from the cell bank of the Shanghai Institute of Cell Biology, Chinese Academy of Sciences (Shanghai, China). HUVECs and HFF-1 were incubated in a low-glucose medium (Gibco, USA) supplemented with 10% fetal bovine serum (CellMax, China) and 1% streptomycin–penicillin solution (Gibco, USA).

### Cell viability analysis

HUVECs/HFF-1 were plated at a density of 5 × 10^3^ cells per well in 96-well plates, with a cell-free group serving as the blank control. The cells were exposed to different concentrations of CAP/CPZ/axitinib. At 24, 48, and, 72 h posttreatment, the cultures were supplemented with 10 μl Cell Counting Kit-8 solution (Dojindo, Japan) per well. Absorbance was then measured at 450 nm using a microplate reader, and the results were calculated by subtracting the blank value from the mean absorbance of each well.

### 5-Ethynyl-2-deoxyuridine (EdU) proliferation assay

EdU proliferation assay was performed using the Cell-Light EdU Apollo567 In Vitro Kit (RiboBio, Guangzhou, Guangdong, China) according to the manufacturer’s instructions. HUVECs were seeded in triplicate in 6-well plates at 1 × 10^5^ cells/ml and cultured with different concentrations of CAP for 48 h. Thereafter, cells were cultured with fresh medium containing 50 μM EdU for 2 h before fixation, neutralization, permeabilization, and Apollo staining. Cell nuclei were subsequently stained with Hoechst33342, and next cells were collected for flow cytometry detection. The ratio of EdU-positive cells was then analyzed using FlowJo software (version 10.8.1).

### Annexin V-fluorescein isothiocyanate (FITC) apoptosis assay

Cell cycle was measured by flow cytometry. HUVECs were harvested, precooled 75% ethanol was added, and the cells were cryopreserved at −20°C for 2 h. Afterwards, the ethanol was discarded, and the cells were washed with PBS at room temperature. They were then stained with Annexin V-FITC and propidium iodide according to the manufacturer’s instructions (Dojindo, Japan) and analyzed using a NoveCyte 3000 flow cytometer (Agilent Technologies, USA). Data were analyzed using FlowJo software (version 10.8.1).

### Cell migration test

HUVECs were seeded in advance on a 6-well culture plate containing 3 ml of complete medium per well. When the confluence reached over 90%, a 200-μl pipette tip was used to create scratch zones by streaking perpendicular to the well. The scratch zones were observed after culture for 0, 8, and 24 h using light microscopy.

### Matrigel tube-formation assay

HUVECs were seeded at a density of 1 × 10^4^ cells per well onto 96-well plates coated with Matrigel (BD Biosciences, USA) and treated with CAP at various concentrations. After incubation at 37°C for 4 or 8 h, tube formation was observed by an inverted microscope (Leica, Germany). The total branching points and total capillary length were counted by ImageJ and Angiogenesis Analyzer software package. Branch points were defined as intersections of ≥3 branches in the tubular network, identified via the Angiogenesis Analyzer plugin, which automatically detects endothelial cell junctions. Total capillary length was calculated as the sum of straight-line distances between branch points along the primary vascular network, determined using the “Analyze > Measure” function in ImageJ, with manual correction for overlapping or misaligned segments. To ensure segmentation accuracy, images were converted to 8-bit grayscale and thresholded using Otsu’s algorithm to distinguish endothelial cells from the Matrigel background.

### Enzyme-linked immunosorbent assay

IL-6 in the HUVEC-cultured medium was detected using a human IL-6 enzyme-linked immunosorbent assay kit (Proteintech; KE00139) according to the manufacturer’s instructions.

### RNA interference

SiRNAs targeting NF-κB (Si-NF-κB #1, 2, and 3, Table S5, see online supplementary material) were obtained from Sangon Biotech (Shanghai, China). Cell transfection was performed according to the manufacturer’s instructions. In brief, HUVECs were transfected with si-NF-κB #1, 2, and 3 or universal negative control siRNAs (Si-Con) using Lipofectamine 3000 (Invitrogen, CA). After incubation for 48 h, the inhibitory efficiency of these siRNAs was verified by q-PCR analysis. The most effective siRNAs (Si-NF-κB#1) were used for further experiments.

### NF-κB inhibitor BAY11-7082 treatment

The pharmacological concentrations (5 μM) and exposure durations (2 h) for NF-κB inhibitor BAY 11-7082 (Selleck Chemicals, USA) were predetermined in the treatment protocol [27, 28].

### Recombinant human IL-6 treatment

One hour pretreatment with 4 ng/ml recombinant human IL-6 (rhIL-6) (MedChemExpress, Shanghai, China) was performed in HUVECs according to the manufacturer’s instructions, and the cells were used in a rescue experiment.

### Outcome assessment

HS severity was assessed by the physician using the Vancouver Scar Scale (VSS) score which measures four parameters: vascularity, pigmentation, pliability, and height [8, 29]. Vascularization is assessed on a scale from 0 (normal) to 3 (purple appearance); pigmentation is classified as 0 for NS color, 1 for hypopigmentation, and 2 for hyperpigmentation; pliability is rated on a scale from 0 (NS) to 5 (contracture that produces deformity), and height is measured on a scale from 0 to 3, where 0 represents a linear shape and 3 represents a scar with a height >5 mm. The subitems are summed to obtain a total score ranging from 0 (representing NS) to 13 (representing the worst imaginable scar).

Poor prognosis refers to the recurrence of HS disease, defined by objective physician assessment as a VSS height score ≥ 2 (HS height ≥ 2 mm) [30].

### Statistical analysis

Data analysis was performed using GraphPad Prism (9.0.0, USA), and R software (version 4.2.2) was utilized for part of the data analyses. Continuous variables are presented as mean ± standard deviation. An independent Student’s *t*-test was applied to compare the means of two groups. One-way analysis of variance (ANOVA) was performed to compare the means of multiple groups with a single factor, and if significant, a post hoc Tukey’s test was used to explore the pairwise differences. For experiments involving two factors (e.g. treatment and time), two-way ANOVA was employed to assess main effects and interaction effects, followed by Tukey’s post hoc test for multiple comparisons where appropriate. *P*-values < 0.05 were considered statistically significant.

## Results

### scRNA-seq reveals cell heterogeneity in the vascular endothelial cells of NS and HS

**Figure 1:**
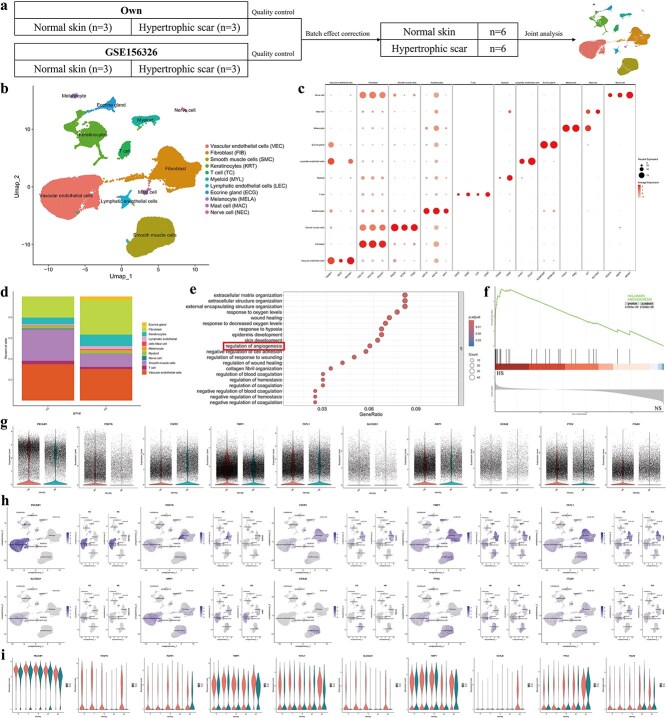
ScRNA-seq reveals the role of vascular endothelial cells in scar formation and development. (**a**) A schematic workflow for scRNA-seq analysis of human NS and HS tissues (*n* = 6 biological replicates per group), integrating data from GSE156326 and our own dataset. (**b**) UMAP plot displaying 79 281 human cells, with 11 distinct cellular clusters identified and colored differently. (**c**) Dotplot visualizing the expression levels and distribution of selected cluster-specific genes, with gene names indicated below. (**d**) Proportions of cell lineages in NS and HS. (**e**) GO biological process enrichment analysis comparing NS and HS. (**f**) GSEA enrichment plot indicating significant upregulation of angiogenesis hallmark gene sets in HS compared to NS (*P*-adj < 0.05, FDR < 0.25). (**g**) Violin plots showing the expression of 10 vascular endothelial cell-specific marker genes in NS and HS. (**h**) UMAP plots depicting the distribution of these 10 marker genes across all cells, and separately in NS and HS. (**i**) Violin plots illustrating the expression of these 10 vascular endothelial cell-specific marker genes within vascular endothelial cell subclusters (clusters C0, C1, C6, C10, and C16) in NS and HS. *ScRNA-seq* single-cell RNA-seq, *NS* normal skin, *HS* hypertrophic scar, *UMAP* uniform manifold approximation and projection, *GO* gene ontology, *GSEA* gene-set enrichment analysis *P-adj* adjusted *P*-value, *FDR* false discovery rate

To investigate the cellular heterogeneity and role of vascular endothelial cells in HS development, we performed scRNA-seq on our own independent biological samples (*n* = 3 NS, *n* = 3 HS) and integrated these data with the public dataset GSE156326 containing three NS and three HS samples as shown in Figure 1a [31]. After quality control, a total of 79 281 cell transcriptomes were obtained (NS: 34 733, HS: 44 548). The filtered data were subjected to PCA to identify integration anchors, followed by batch effect correction using the IntegrateLayers function with the Harmony algorithm. In this analysis, 11 cell types were identified in combination with the characteristic gene expression of each cluster (Figure 1b). These cell types included vascular endothelial cells (marked by TM4SF1, PECAM1, and SELE), smooth muscle cells (marked by TAGLN, ACTA2, and TPM2), and fibroblasts (marked by COL1A1, COL1A2, and COL3A1) (Figure 1c). Based on hierarchical clustering and established lineage-specific marker genes, we calculated the relative proportions of the 11 distinct cell clusters in both the NS and HS samples (Figure 1d). GO pathway analysis revealed that various pathways including regulation of angiogenesis were affected in the HS samples (Figure 1e). Furthermore, GSEA identified that angiogenesis pathways were enriched in the HS samples (Figure 1f).

Next, we identified the expression of 10 specific genes associated with angiogenesis (PECAM1 [32], POSTN [33], FGFR1 [34], TIMP1 [35], FSTL1 [36], SLCO2A1 [37], NRP1 [38], KCNJ8 [39], PTK2 [40], and ITGAV [41]) between the NS and HS. We found that those angiogenesis-associated genes were significantly increased in the vascular endothelial cells of the HS group (Figure 1g and h). Angiogenesis is closely related to vascular endothelial cells; thus, we focused on this vascular endothelial cell subpopulation. Hierarchical cluster analysis suggested that vascular endothelial cells from NS and HS samples could be divided into five subpopulations. We calculated the expression levels of angiogenesis-associated genes in each of the five vascular endothelial cell subpopulations and those genes were upregulated in the HS samples (Figure 1i). At the same time, we observed that Cluster 16, representing vascular endothelial cells, exhibited significantly higher expression levels of angiogenesis-related factors compared to other subclusters. Collectively, these results suggest that excessive angiogenesis occurs, and vascular endothelial cells undergo significant changes during HS formation.

### Bulk RNA-seq analysis suggests that the formation of HS is closely related to angiogenesis and may be associated with the TRPV1 channel

**Figure 2:**
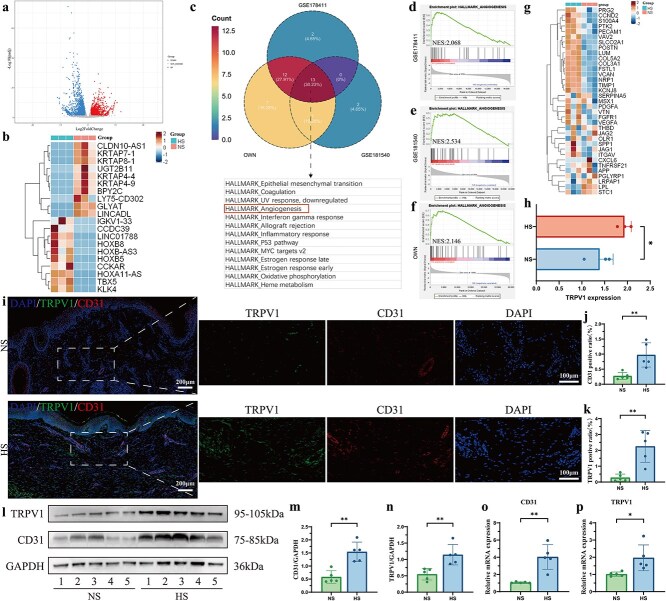
Bulk RNA-seq analysis suggests that the formation of HS is linked to angiogenesis, which may be associated with the TRPV1 channel. (**a**) Volcano plots of DEGs between NS (*n* = 3 biologically independent samples) and HS (*n* = 3 biologically independent samples) bulk RNA-seq samples. (**b**) Heatmap shows clustering of DEGs between NS and HS; upregulated genes are highlighted in red and downregulated genes in blue. (**c**) Venn diagram displaying significantly upregulated (*P*-adj < 0.05, FDR < 0.25) GSEA enrichment plots in HS compared to NS across bulk RNA-seq datasets GSE178411, GSE181540, and our dataset, with 13 overlapping angiogenesis hallmark gene sets identified. (**d**–**f**) GSEA enrichment plots indicating significant up-regulation of angiogenesis hallmark gene sets in HS compared to NS in datasets GSE178411 (**d**), GSE181540 (**e**), and our RNA-seq dataset (**f**) (*P*-adj < 0.05, FDR < 0.25). (**g**) Heatmap demonstrating expression levels of angiogenesis-related genes between NS and HS, with selected genes listed on the right; upregulated genes in red and downregulated in blue. (**h**) Expression levels of TRPV1 in NS and HS bulk RNA-seq samples. (**i**–**k**) Representative images of CD31 and TRPV1 coimmunostaining (**i**), with quantification of CD31 (**j**) and TRPV1 (**k**) positive ratios in NS and HS samples; *n* = 5 biologically independent samples. Scale bar: 100 μm & 200 μm. (**l**–**n**) Expression levels of CD31 (**m**) and TRPV1 (**n**) in NS and HS samples measured by western blotting; *n* = 5 biologically independent samples. (**op**) mRNA expression levels of CD31 (**o**) and TRPV1 (**p**) in NS and HS samples. *n* = 5 biologically independent samples. Data are presented as the mean ± SD. ^*^P < 0.05, ^**^P < 0.01. *HS* hypertrophic scar, *DEGs* differentially expressed genes, *NS* normal skin, *P*-*adj* adjusted *P*-value, *FDR* false discovery rate, *GSEA* gene-set enrichment analysis, *NES* normalized enrichment score

To validate the correlation between angiogenesis and HS in single-cell RNA sequencing and to further elucidate the mechanism of angiogenesis, we performed bulk RNA sequencing of three NS samples and three HS samples. We identified 3837 DEGs between the NS samples and the HS samples of which 2167 genes were upregulated and 1670 were downregulated (|FC| ≥ 1, *P* < 0.05) (Figure 2a). GO and KEGG pathway analysis found that various pathways including regulation of angiogenesis and VEGF production were affected in HS samples (Figure S1a and b, see online supplementary material). Besides, significantly DEGs are illustrated in a heatmap (Figure 2b). To determine the generally affected gene sets among the genes whose expression differed between the NS samples and the HS samples, we performed GSEA analysis on our own and another two bulk RNA sequencing datasets (GSE178411 and GSE181540) and compared the results using a Venn diagram (Figure 2c). We found 13 simultaneously overlapping GSEA gene sets and the hallmark of angiogenesis was among them. To be specific, GSEA revealed a significant positive enrichment of angiogenesis (NES = 2.068 in GSE1784111, NES = 2.534 in GSE181540, and NES = 2.146 in our own dataset) (Figure 2d–f). Additionally, we demonstrated that angiogenesis-related genes are highly enriched in HS compared with NS by a heatmap illustration (Figure 2g).

**Figure 3:**
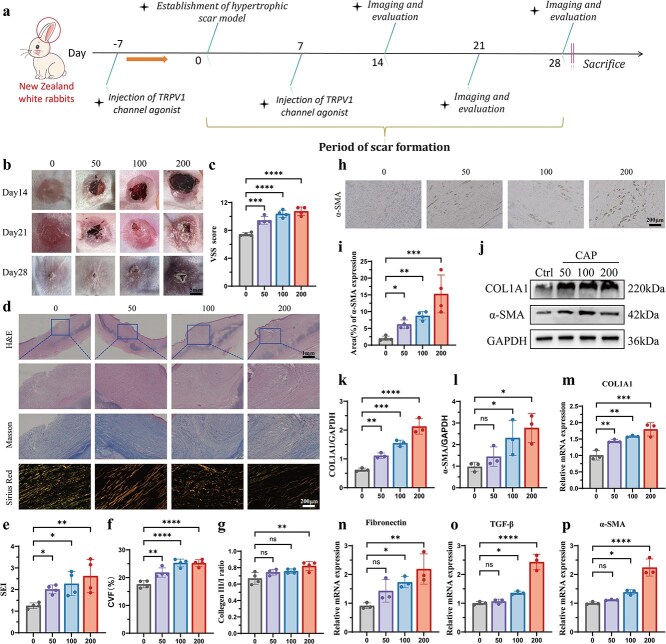
Activation of the TRPV1 channel promotes scar formation and fibrosis *in vivo*. (**a**) Schematic workflow for establishing and evaluating the rabbit ear HS model. CAP, a TRPV1 channel agonist, was injected at doses of 0, 50, 100, and 200 mg/kg; *n* = 4 biologically independent samples. (**b**) Representative photographs of rabbit ear wounds treated with different doses at days 14, 21, and 28. Scale bar: 5 mm. (**c**) VSS scores of HS tissues in the 0, 50, 100, 200 mg/kg groups at day 28. *n* = 4 biologically independent samples. (**d**) Representative images of H&E staining, Masson staining, and Sirius red staining of HS tissues in all groups at day 28. Scale bar: 1 mm & 200 μm. (**e**–**g**) Quantitative analysis of SEI (**e**), CVF (**f**), and type III/I collagen ratio (**g**) in HS tissues across the four groups at day 28. *n* = 4 biologically independent samples. (**hi**) Representative α-SMA immunostaining images (**h**) and quantitative analysis (**i**) of HS tissues in all groups at day 28. *n* = 4 biologically independent samples. Scale bar: 200 μm. (**j**–**l**) Expression levels of COL1A1 (**k**) and α-SMA (**l**) in HS tissues across the four groups at day 28, measured by western blotting. *n* = 3 biologically independent sample. (**m**–**p**) Expression levels of COL1A1 (**m**), fibronectin (**n**), TGF-β (**o**), and α-SMA (**p**) in HS tissues across the four groups at day 28, measured by q-PCR. *n* = 3 biologically independent samples; data are presented as the mean ± SD. ns, not significant, ^*^P < 0.05, ^**^P < 0.01, ^***^P < 0.001, ^****^P < 0.0001. *HS* hypertrophic scar, *CAP* capsaicin, *VSS* Vancouver Scar Scale, *SEI* scar evaluation index, *CVF* collagen volume fraction

According to scRNA-seq and bulk RNA-seq analyses, we identified that angiogenesis mediated by vascular endothelial cells has a close connection with HS compared to NS. As mentioned before, the main ingredient in chili peppers is CAP, and previous studies have shown that consuming spicy foods can exacerbate HS symptoms. CAP is a TRPV1 channel agonist, and the TRPV1 expression level in bulk RNA-seq was actually upregulated in HS compared with that in NS (Figure 2h). Furthermore, we conducted IF staining, western blotting, and q-PCR on skin tissues derived from NS and HS. IF staining revealed aggregated and heightened expression of CD31 (a vascular endothelial cell marker) and TRPV1 in the dermis of HS compared to NS (Figure 2i–k). Western blotting confirmed upregulated CD31 and TRPV1 protein levels in the HS group (Figure 2l–n). Finally, q-PCR analysis validated increased mRNA expression of CD31 and TRPV1 in HS tissues (Figure 2o and p).

### *In vivo* activation of the TRPV1 channel promotes fibrosis and scar formation

To explore the association between the activation of TRPV1 channel and HS, we employed the rabbit ear model which resembles a human HS (Figure 3a). The rabbits were divided into four groups randomly with 0, 50, 100, 200 mg/kg of the TRPV1 channel agonist CAP injected, and the HS models were successfully established on day 28 (Figure 3b). HS exhibited red coloration and raised scar surfaces in the three CAP-injected groups, and as the concentration increased, their VSS scores also increased (Figure 3b and c). Then we performed various histological analyses to observe the hypertrophic features (Figure 3d). Through hematoxylin and eosin (H&E) staining, we observed that the introduction of CAP thickened and raised the dermis, which was further demonstrated by the SEI analysis (Figure 3d and e). Masson staining results also revealed that the collagen fibers (stained blue) in the dermis treated with CAP were significantly increased compared to that in the control group (Figure 3d and f). Additionally, the ratio of type III to I collagen calculated from images of Sirius Red staining images was lifted in the 200-injected group compared to the control group (Figure 3d and g). These histological results indicate that *in vivo* activation of the TRPV1 channel could promote scar formation.

To further investigate the condition of scar fibrosis, we performed immunohistochemical staining, western blotting, and q-PCR on rabbit HS tissues to assess fibrosis-related indices. The expression level of α-SMA was positively correlated with CAP concentrations as indicated by the results of the α-SMA immunohistochemical staining results (Figure 3h and i). In addition, western blotting confirmed higher Collagen I expression in the three CAP-injected groups than in the control group (Figure 3j and k) and higher α-SMA expression in the 200-injected group than in the control group (Figure 3j and l). Finally, tissue q-PCR analysis showed that the mRNA expression level of COL1A1 was positively correlated with CAP concentrations (Figure 3m), while the mRNA expression level of fibronectin, TGF-β, and α-SMA was increased in the 100-injected and 200-injected groups compared to the control group (Figure 3n–p). These findings suggest that activation of the TRPV1 channel can significantly promote scar formation and fibrosis *in vivo*.

### *In vivo* activation of the TRPV1 channel promotes scar angiogenesis

**Figure 4:**
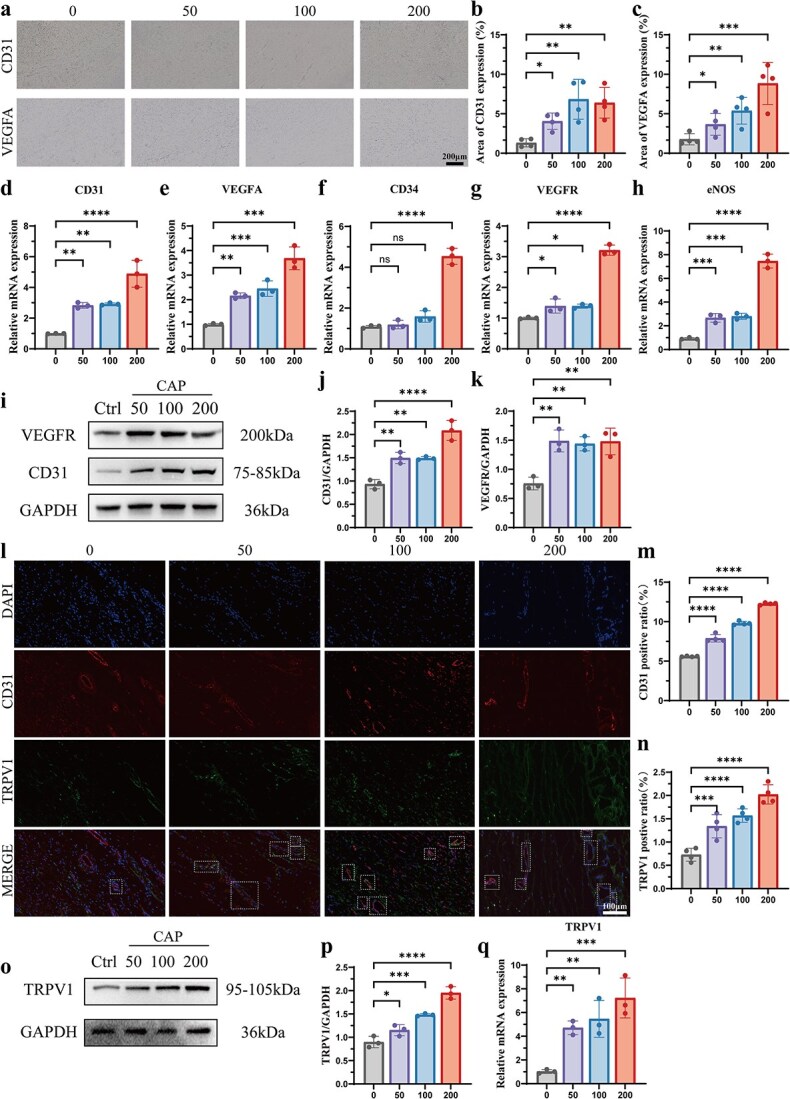
Activation of the TRPV1 channel promotes scar vascularization *in vivo*. (a–c) Representative immunostaining images for CD31 and VEGFA (**a**) and corresponding quantitative analyses (**bc**) of HS tissues across four groups at day 28. *n* = 4 biologically independent samples. Scale bar: 200 μm. (**d**–**h**) Expression levels of CD31 (**d**), CD34 (**e**), VEGFA (**f**), VEGFR (**g**), and eNOS (**h**) in HS tissues were measured by q-PCR across the four groups. *n* = 3 biologically independent samples. (**i**–**k**) Expression levels of VEGFR (**j**) and CD31 (**k**) in HS tissues were assessed using western blotting across the four groups at day 28. *n* = 3 biologically independent samples. (**l**–**n**) Representative coimmunostaining images for CD31 and TRPV1 (**l**) with quantification of the positive ratios for CD31 (**m**) and TRPV1 (**n**) in the four groups. *n* = 4 biologically independent samples. Scale bar: 100 μm. (**op**) TRPV1 expression levels in HS tissues were measured by western blotting across the four groups at day 28. *n* = 3 biologically independent samples. (**q**) TRPV1 expression levels in HS tissues were also measured by q-PCR across the four groups. *n* = 3 biologically independent samples; data are presented as the mean ± SD; ns, not significant, ^*^P < 0.05, ^**^P < 0.01, ^***^P < 0.001, ^****^P < 0.0001. *HS* hypertrophic scar, *CAP* capsaicin, *q-PCR* quantitative real time PCR

To further investigate the effect of TRPV1 channel activation on scar angiogenesis, *in vivo* angiogenesis-related indices were initially evaluated using immunohistochemical staining, western blotting, and q-PCR. From the immunohistochemical staining analysis, we found that the expression level of CD31 positively correlated with CAP concentrations (Figure 4a and b). Similarly, VEGFA staining was widely distributed in the three CAP-injected groups (Figure 4a and c). Furthermore, we evaluated the mRNA expression levels of endothelial cell markers (CD31, VEGFA, CD34, VEGFR, eNOS) by q-PCR. Consistent with the immunostaining results, CD31 and VEGFA mRNA expression levels were significantly increased in the CAP-injected groups (Figure 4d and e). Additionally, the CD34 mRNA expression level increased from 1.09 ± 0.07 (control group) to 4.54 ± 0.40 (200-injected group) (Figure 4f). Similarly, the mRNA expression level of VEGFR and eNOS was increased in the three CAP-injected groups than in the control group (Figure 4g and h). Similar trends were also observed via western blotting, while the protein levels of CD31 and VEGFR were upregulated in rabbit HS tissues after CAP injection (Figure 4i–k).

Complementary, we performed co-IF staining for CD31 and TRPV1 to confirm the relationship between TRPV1 channel activation and angiogenesis (Figure 4l). We observed that more CD31-positive cells expressed TRPV1 as CAP concentration increased (Figure 4m and n). Finally, we verified the increased expression of TRPV1 after CAP injection using western blotting (Figure 4o and p), while q-PCR also showed a similar trend (Figure 4q). In conclusion, our findings strongly support that CAP injection could activate TRPV1 channel to promote scar fibrosis and angiogenesis *in vivo*.

### *In vivo* ablation of the TRPV1 channel could relieve scar fibrosis and angiogenesis induced by capsaicin

**Figure 5:**
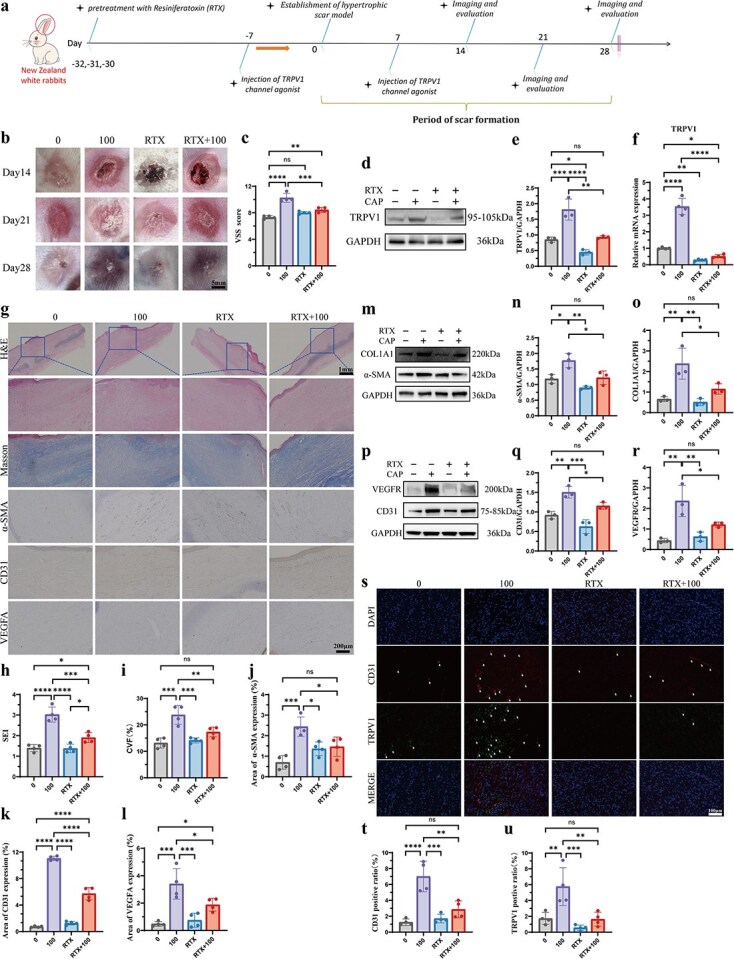
Ablation of the TRPV1 channel alleviates scar fibrosis and angiogenesis induced by CAP *in vivo*. (**a**) Schematic workflow of the rabbit ear HS model establishment and evaluation; subcutaneous injections of RTX result in systemic ablation of the TRPV1 channel; four groups were divided: no treatment (0), 100 mg/kg CAP injected (100), RTX pretreated, RTX pretreated with 100 mg/kg CAP injected (RTX + 100). *n* = 4 biologically independent samples. (**b**) Representative photographs of rabbit ear wounds with different treatments at days 14, 21, and 28. Scale bar: 5 mm. (**c**) VSS scores of the HS tissues in the 0, 100, RTX, and RTX + 100 groups at day 28. *n* = 4 biologically independent samples. (**de**) TRPV1 expression levels in HS tissues of the four groups at day 28, measured by western blotting. *n* = 3 biologically independent samples. (**f**) TRPV1 expression levels in HS tissues of the four groups were measured by q-PCR. *n* = 4 biologically independent samples. (**g**) Representative images of H&E staining, Masson staining, α-SMA immunostaining, CD31 immunostaining, and VEGFA immunostaining of HS tissues in the four groups at day 28. Scale bar: 200 μm. (**hi**) Quantitative analysis of SEI (**h**) and CVF (**i**) of HS tissues in the four groups at day 28. *n* = 4 biologically independent samples. (**j**–**l**) Quantitative analysis of the percentage of α-SMA-positive areas (j), CD31-positive areas (k), and VEGFA-positive areas (l) in the four groups at day 28. *n* = 4 biologically independent samples. (**m**–**o**) COL1A1 and α-SMA expression levels in HS tissues of the four groups at day 28, measured by western blotting. *n* = 3 biologically independent samples. (**p**–**r**) VEGFR and CD31 expression levels in HS tissues of the four groups at day 28, measured by western blotting. *n* = 3 biologically independent samples. (**s**–**u**) Representative coimmunostaining images for CD31 and TRPV1 (**s**) with quantification of the CD31 (**t**) and TRPV1 (**u**) positive ratios in the four groups. *n* = 4 biologically independent samples. Scale bar: 100 μm. Data are presented as the mean ± SD; ns, not significant, ^*^P < 0.05, ^**^P < 0.01, ^***^P < 0.001, ^****^P < 0.0001. *CAP* capsaicin, *RTX* resiniferatoxin, *VSS* Vancouver Scar Scale, *SEI* scar evaluation index, *CVF* collagen volume fraction

As we found that activation of the TRPV1 channel could promote scar fibrosis and angiogenesis *in vivo*, we further employed a rabbit ear model to explore whether RTX (an ultrapotent TRPV1 agonist known to induce long-term desensitization of TRPV1) can ablate TRPV1 afferent fibers and abolish the functional responses of HS induced by TRPV1 agonist CAP [24, 25]. Rabbits were randomly divided into four groups: (**a**) control group (no treatment), (**b**) 100 group (100 mg/kg CAP injected 1 week prior to the establishment of the HS model), (**c**) RTX group (continuous gradient injection of RTX 1 month prior to the establishment of the HS model), and (**d**) RTX + 100 group (continuous gradient injection 1 month prior to the establishment of the HS model and 100 mg/kg CAP injected 1 week prior to the establishment of HS models). We monitored scar formation for 4 weeks, after which we collected samples for evaluation (Figure 5a). HS models were successfully re-established on day 28 and we found that the 100 group exhibited greater redness, more convexity, and a higher VSS score than the other three groups did (Figure 5b and c). Besides, we verified that TRPV1 expression was significantly downregulated in the RTX and RTX + 100 group as shown by western blotting and q-PCR demonstrating that TRPV1 expression is ablated following the *in vivo* injection of RTX (Figure 5d–f).

To further investigate the effect of TRPV1 ablation on scar fibrosis and angiogenesis, fibrosis-related markers and angiogenesis-related indicators were initially evaluated via immunohistochemical staining, western blotting, and q-PCR. Through H&E staining, we observed that RTX intervention reduced scar thickening caused by CAP injection, as further demonstrated by the SEI analysis (Figure 5g and h). Masson staining results also revealed that the collagen fibers in the dermis treated with CAP were significantly increased compared to that in the other three groups (Figure 5g and i). The expression level of the fibrosis marker α-SMA in the RTX and RTX + 100 groups was markedly reduced compared to the 100 group (Figure 5g and j). Similar trends were observed for the expression levels of angiogenesis-marker CD31 and VEGFA (Figure 5g). However, we also found that CD31 and VEGFA expression in the RTX + 100 group was slightly increased from the control group, indicating that RTX ablation reduces CAP-induced scar angiogenesis without completely eliminating it (Figure 5k and l).

In addition, western blotting confirmed higher Collagen I and α-SMA expression in the 100 group than in control, RTX, and RTX + 100 group (Figure 5m–o), while the mRNA expression levels of α-SMA, TGF-β, and COL1A1 were lower in rabbit HS tissues with RTX injected compared to those injected only with CAP (Figure S2a–c, see online supplementary material). For CD31 and VEGFR expression evaluated by western blotting, levels were reduced in the RTX group compared to the 100 group, but there was no statistically significant difference in the RTX + 100 group compared to the 100 group, despite a decrease (Figure 5p–r). Furthermore, we evaluated the mRNA expression level of endothelial cell markers (CD31, CD34, and VEGFR) by q-PCR analysis. Similar to the results of fibrosis-marker mRNA expression, the mRNA expression level of CD31, CD34, and VEGFR was decreased in the RTX and RTX + 100 groups than in the 100 group (Figure S2d–f, see online supplementary material).

Complementary, we performed co-IF staining of CD31 and TRPV1 to confirm the relationship between TRPV1 ablation and angiogenesis (Figure 5s). We observed that the number of CD31-positive cells decreased and that these cells expressed lower levels of TRPV1 following RTX injection, regardless of whether they received subsequent CAP injections (Figure 5t and u). These results suggest that RTX ablation significantly alleviates scar fibrosis and angiogenesis due to CAP-induced TRPV1 activation.

### Local injection of the TRPV1 blocker capsazepine and the antiangiogenic drug axitinib alleviated HS formation induced by capsaicin *in vivo*

Since we demonstrated that systemic activation of the TRPV1 channel by CAP promotes HS formation, we hypothesized that local inhibition of TRPV1 using the antagonist CPZ could suppress scar development. To validate this, we first conducted *in vitro* cytotoxicity assays on both HUVECs and HFF-1 cells to determine the IC_50_ values of CPZ. The calculated IC_50_ values were 114.5 μM (43.16 μg/ml) for HUVECs and 73.2 μM (27.6 μg/ml) for HFF-1 cells (Figure S3a, see online supplementary material). Notably, the IC_50_ value for HUVECs was close to 40 μg/ml, which served as the rationale for selecting this concentration in subsequent *in vivo* experiments. Then, 100 mg/kg CAP was administered subcutaneously to the dorsal thoracic region of New Zealand White rabbits 1 week prior to the establishment of HS model. The animals were randomly divided into four groups (*n* = 4/group) and received local injections of 0, 40 μg/ml, 400 μg/ml, or 4 mg/ml CPZ at 2 and 3 weeks before surgery (Figure S3b, see online supplementary material). On day 28, the HS models were successfully established, and histological evaluation revealed that the control group exhibited significantly greater redness, convexity, and a higher VSS score compared to the CPZ-treated groups (Figure S3c and d, see online supplementary material). To further investigate the antifibrotic and antiangiogenic effects of TRPV1 inhibition, we evaluated fibrosis-related markers and angiogenesis-related indicators via immunohistochemical staining. H&E staining demonstrated that CPZ treatment reduced scar thickening, with 400 μg/ml CPZ showing the most pronounced effect as evidenced by the SEI analysis (Figure S3e and f, see online supplementary material). Masson staining confirmed a significant reduction in dermal collagen deposition in the CPZ-treated groups compared with that in the control group (Figure S3e and g, see online supplementary material). Immunohistochemical analysis revealed that the expression levels of fibrosis markers α-SMA and VEGFA were markedly decreased in all CPZ-treated groups compared to the control (Figure S3h and i, see online supplementary material). Additionally, co-immunofluorescence staining of CD31 and TRPV1 showed a reduction in CD31-positive cells and TRPV1 expression following CPZ administration (Figure S3j–l, see online supplementary material). These findings collectively indicate that early local application of CPZ significantly attenuated CAP-induced hypertrophic scarring, with 400 μg/ml demonstrating the most robust efficacy. Importantly, these results represent the first validation of this dosing strategy specifically for HS models. However, the 4 mg/ml group may have exhibited efficacy primarily due to significant cytotoxicity, as this concentration represents ~100-fold the IC50 value for HUVECs and HFF-1, potentially leading to non-specific tissue damage rather than selective TRPV1 inhibition.

Complementarily, we included new experiments in which the VEGFR inhibitor axitinib (an antiangiogenic drug) was used to verify the relationship between TRPV1 activation and angiogenesis. We conducted *in vitro* cytotoxicity assays for axitinib on HUVECs and HFF-1 cells. The IC_50_ values were determined to be 211.6 μM (81.78 μg/ml) and 171.3 μM (66.2 μg/ml), respectively (Figure S4a, see online supplementary material). Then the rabbits were randomly divided into four groups: (1) the control group (no treatment), (2) the CAP group (100 mg/kg CAP systematically administered 1 week prior to HS model establishment), (3) the axitinib group (local injection of 1.25 mg/ml axitinib at 2 and 3 weeks before surgery), and (4) the CAP + axitinib group (100 mg/kg CAP pretreated with local injection of axitinib at 2 and 3 weeks before surgery). Notably, this concentration range (1.25 mg/ml) is well above the IC_50_ threshold, validating its safety for scar tissue applications based on prior study [42]. Scar formation was monitored for 4 weeks, and samples were collected for evaluation (Figure S4b, see online supplementary material). On day 28, the HS models were successfully re-established, and we found that the CAP group exhibited greater redness, more convexity, and a higher VSS score compared to the CAP + axitinib group, while the axitinib group and Control group showed no significant differences (Figure S4c and d, see online supplementary material). Additionally, we observed through H&E staining that axitinib alone resulted in a modest reduction in scar thickness and SEI score compared to the Ctrl group, whereas axitinib administration significantly attenuated the CAP-induced scar thickening and SEI elevation (Figure S4e and f, see online supplementary material). Masson staining results demonstrated that the collagen fibers in the CAP + axitinib group were significantly reduced compared to those in the CAP group (Figure S4e and g, see online supplementary material). Consistent with this, the expression level of the fibrosis marker α-SMA was also markedly decreased in the CAP + axitinib group compared to the CAP group (Figure S4e and h, see online supplementary material). Notably, VEGFA expression in the axitinib group was significantly lower than in the control group, and the same reduction was observed in the CAP + axitinib group compared to the CAP group, confirming the efficacy of axitinib in inhibiting VEGFA-mediated angiogenesis (Figure S4e and i, see online supplementary material). Furthermore, we performed co-IF staining of CD31 and TRPV1 to investigate the effects of axitinib on TRPV1 expression and angiogenesis (Figure S4j, see online supplementary material). The results showed that CD31-positive cells were significantly reduced in the axitinib-treated groups compared to the control group, consistent with the known antiangiogenic activity of axitinib, which inhibits VEGF signaling. However, axitinib did not significantly downregulate TRPV1 expression in the treated tissues, suggesting that its antiscarring effects may primarily operate through VEGF-dependent pathways rather than direct modulation of TRPV1 (Figure S4k and l, see online supplementary material). While the dosing of axitinib was pharmacologically effective and did not induce overt tissue toxicity in our *in vivo* experiments, its application in HS angiogenesis currently lacks a solid mechanistic foundation. To advance precision medicine, systematic PK-PD modeling integrating VEGF signaling kinetics is essential to define optimal therapeutic regimens.

**Figure 6:**
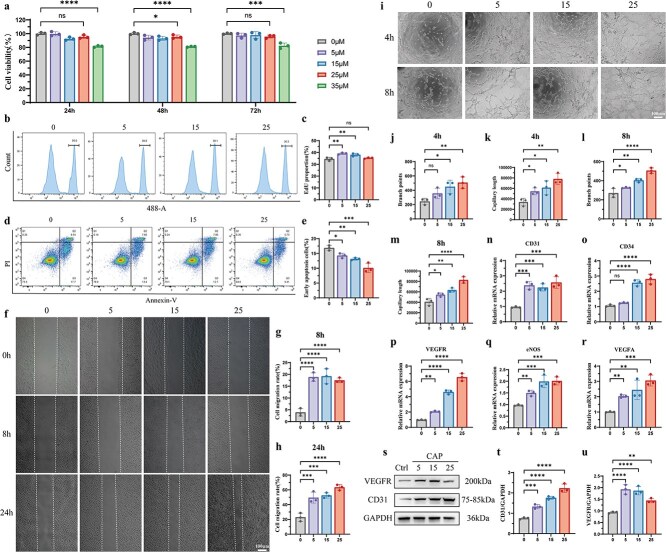
The biological properties and functions of HUVECs are regulated by the activation of the TRPV1 channel *in vitro*. (**a**) Cell viability of HUVECs was assessed using the CCK8 assay with different concentrations of the TRPV1 channel agonist CAP (0, 5, 15, 25, and 35 μM) after incubation for 24, 48, and 72 h. *n* = 3 independent experiments. (**bc**) Cell proliferation ability was measured using the EdU test. The expression of EdU in HUVECs was detected after treatment with CAP (0, 5, 15, and 25 μM) for 24 h. *n* = 3 independent experiments. (**de**) Apoptosis was measured by flow cytometry with Annexin V-FITC/PI staining; HUVECs were treated with CAP (0, 5, 15, and 25 μM) and the Q3 quadrant represented early apoptotic cells. *n* = 3 independent experiments. (**f**–**h**) Migration experiment was assessed using the cell scratch test. After creating a scratch in HUVECs (0 h), cells were treated with CAP at the indicated concentrations; representative images (**f**) and quantitative analyses (**gh**) of migration are shown at 8 and 24 h. Scale bar: 100 μm. *n* = 3 independent experiments. (**i**–**m**) Representative optical images (**i**) of *in vitro* tubular network formed by HUVECs treated with CAP at the indicated concentrations; quantitative analyses of branch points and capillary length are shown at 4 (**jk**) and 8 h (**lm**). *n* = 3 independent experiments. Scale bar: 100 μm. (**n**–**r**) Expression levels of CD31 (**n**), CD34 (**o**), VEGFR (**p**), eNOS (**q**), and VEGFA (**r**) in HUVECs treated with CAP at the indicated concentrations were measured by q-PCR. *n* = 3 independent experiments. (**s**–**u**) Expression levels of CD31(**t**) and VEGFR(**u**) in HUVECs treated with CAP at the indicated concentrations were measured by western blotting. *n* = 3 independent experiments; data are presented as the mean ± SD. ns, not significant. ^*^P < 0.05, ^**^P < 0.01, ^***^P < 0.001, ^****^P < 0.0001. *HUVECs* human umbilical vein endothelial cells, *CCK8* cell Counting Kit 8, *CAP* capsaicin, *EdU* 5-Ethynyl-2-deoxyuridine, *PI* propidium iodide, *q-PCR* quantitative real time PCR

### *In vitro* activation of the TRPV1 channel regulates the biological properties and functions of HUVECs

We found that *in vivo* activation of the TRPV1 channel by CAP promotes scar angiogenesis, so we next evaluated the effect of CAP-induced activation of the TRPV1 channel *in vitro* via HUVECs. A Cell Counting Kit-8 assay was used to examine the cytotoxicity of cells incubated with CAP. The results showed that when the concentration was below 35 μM, CAP did not affect cell viability (Figure 6a). EdU assays revealed that the proliferation rates of HUVECs treated with 5 μM and 15 μM CAP were significantly increased, while the 25 μM CAP group showed no significant differences in proliferation (Figure 6b and c). Furthermore, flow cytometry analysis indicated a significant reduction in apoptosis with increasing CAP concentration (Figure 6d and e). In addition, the results of the wound-healing experiment showed that CAP significantly promoted the migration of HUVECs (Figure 6f–h). In brief, *in vitro* activation of TRPV1 by CAP promoted the viability and migration but reduced the apoptosis of HUVECs.

To investigate the association between the TRPV1 channel and angiogenesis *in vitro*, we performed a Matrigel tube-formation assay, which demonstrated a significantly enhanced angiogenic capacity of CAP-cultured HUVECs, based on branch points and tube capillary length (Figure 6i–m). We also assessed the mRNA expression of angiogenesis-related markers (CD31, CD34, VEGFR, eNOS, VEGFA) which indicated a remarkable increase in HUVECs treated with CAP (Figure 6n–r). The western blotting results showed similar trends, with the expression of CD31 and VEGFR differentially elevated upon TRPV1 activation (Figure 6s–u). These results suggest that TRPV1 channel activation significantly regulates the biological properties and angiogenesis functions of HUVECs. Moreover, our data demonstrate that CAP exerts substantially potent effect on vascular tube formation compared to cell migration, which may be attributed to distinct signaling pathways in different biological contexts.

### TRPV1 channel activation regulates angiogenesis in HUVECs via the IL-6/STAT3 signaling pathway

**Figure 7:**
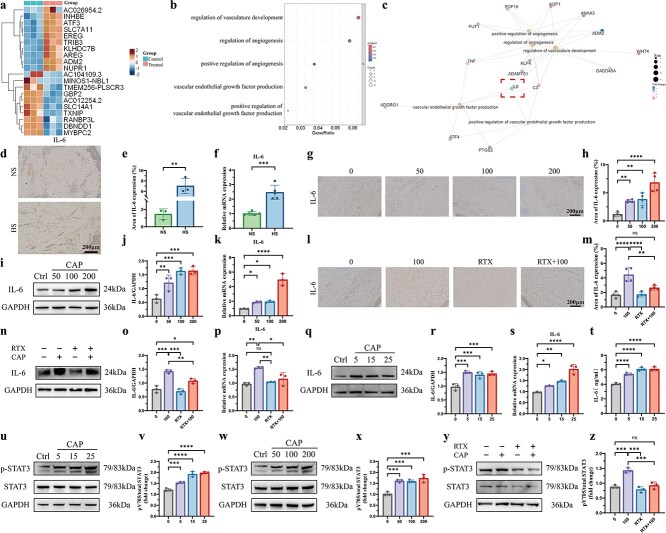
TRPV1 channel activation regulates the angiogenesis of HUVECs via the IL-6/STAT3 signaling pathway. (**a**) Heatmap showing prominently upregulated and downregulated genes between normal HUVECs (control group, *n* = 3 biologically independent samples) and HUVECs treated with the TRPV1 channel agonist CAP (treated group, *n* = 3 biologically independent samples). Selected genes related to RNA transcriptome sequencing are shown on the right, with upregulated genes highlighted in red and downregulated genes in blue. (**b**) Enrichment plots of GO terms related to angiogenesis between the control and treated group using DEGs. (**c**) Connection plot illustrating the interrelation among angiogenesis terms and genes, highlighting the IL-6 gene linked to angiogenesis-related GO terms. (**de**) Representative IL-6 immunostaining images (**d**) and quantitative analysis (**e**) comparing NS samples and HS samples. *n* = 3 biologically independent samples. Scale bar: 200 μm. (**f**) The expression level of IL-6 between NS samples and HS samples measured by q-PCR. *n* = 5 biologically independent samples. (**gh**) Representative IL-6 immunostaining images (**g**) and quantitative analysis (**h**) of rabbit ear HS tissues in the 0. 50, 100, and 200 mg/kg CAP groups at day 28. *n* = 4 biologically independent samples. Scale bar: 200 μm. (**ij**) IL-6 expression levels in HS tissues across the 0. 50, 100, and 200 mg/kg groups at day 28, measured by western blotting. *n* = 3 biologically independent samples. (**k**) IL-6 expression levels in HS tissues in the 0, 50, 100, and 200 mg/kg groups measured by q-PCR. *n* = 3 biologically independent samples. (**lm**) Representative IL-6 immunostaining images (**l**) and quantitative analysis (**m**) of rabbit ear HS tissues in the 0, 100, RTX, and RTX + 100 groups (0: no treatment, 100: 100 mg/kg CAP injected, RTX: RTX pretreated, RTX + 100: RTX pretreated with 100 mg/kg CAP injected) at day 28. *n* = 4 biologically independent samples. Scale bar: 200 μm. (**no**) IL-6 expression levels of HS tissues in the 0, 100, RTX, and RTX + 100 groups at day 28, measured by western blotting. *n* = 3 biologically independent samples. (**p**) IL-6 expression levels in HS tissues in the 0, 100, RTX, and RTX + 100 groups measured by q-PCR. *n* = 3 biologically independent samples. (**qr**) IL-6 expression levels in HUVECs treated with 0, 5, 15, and 25 μM CAP, measured by western blotting. *n* = 3 independent experiments. (**s**) IL-6 expression levels in HUVECs treated with CAP at the indicated concentrations, measured by q-PCR. *n* = 3 independent experiments. (**t**) Secreted IL-6 expression levels from HUVECs treated with CAP at the indicated concentrations, measured using an ELISA-based kit. *n* = 3 independent experiments. (**uv**) Representative blots (**u**) and quantitative analysis (**v**) of pY705-STAT3 over total STAT3 in HUVECs treated with 0, 5, 15, and 25 μM CAP. *n* = 3 independent experiments. (**wx**) Representative blots (**w**) and quantitation analysis (**x**) of pY705-STAT3 over total STAT3 in HS tissues (0, 50, 100, and 200 mg/kg groups). *n* = 3 biologically independent samples. (**yz**) Representative blots (**y**) and quantitation analysis (**z**) of pY705-STAT3 over total STAT3 in HS tissues (0, 100, RTX, and RTX + 100 groups). *n* = 3 biologically independent samples. Data are presented as the mean ± SD. ns, not significant, ^*^P < 0.05, ^**^P < 0.01, ^***^P < 0.001, ^****^P < 0.0001. *HUVECs* human umbilical vein endothelial cells, *CAP* capsaicin, *GO* gene ontology, *DEGs* differentially expressed genes, *NS* normal skin, *HS* hypertrophic scar, *q-PCR* quantitative real time PCR, *RTX* resiniferatoxin

To further elucidate the mechanism by which TRPV1 affects the biological properties and angiogenesis of HUVECs, RNA transcriptome sequencing was performed to identify the downstream genes regulated by TRPV1. Prominently upregulated and downregulated genes between normal HUVECs and CAP-treated HUVECs are shown in a heatmap (Figure 7a). GO and KEGG pathway analyses revealed that various pathways were affected in HUVECs after treatment with CAP (Figure S5a and b, see online supplementary material). In addition, we extracted the top five terms from the GO analysis along with their associated genes for PPI network analysis and mapped the enriched interaction network of these terms (Figure S5c and d, see online supplementary material).

We then detected that the GO terms related to angiogenesis were enriched in HUVECs treated with the TRPV1 agonist CAP using the DEGs (Figure 7b). A connection plot showing the interrelations among the angiogenesis terms and genes was created, highlighting that the IL-6 gene is linked to the GO terms related to angiogenesis (Figure 7c). These findings suggest that IL-6 may be an important downstream gene affecting angiogenesis following TRPV1 activation.

To further elucidate the role of IL-6 in HS, we examined the expression pattern of IL-6 using immunohistochemical staining and q-PCR. The results indicated increased protein levels (Figure 7d and e) and mRNA expression (Figure 7f) of IL-6 in human HS compared with those in NS. We then examined the expression of IL-6 in rabbit ear HS tissues treated with CAP using immunohistochemistry (Figure 7g and h), western blotting (Figure 7i and j), and q-PCR (Figure 7k) confirming a significant increase in IL-6 expression. In addition, we assessed the IL-6 expression in rabbit ear HS tissues with the ablation of the TRPV1 channel. Using immunohistochemical staining (Figure 7l and m), western blotting (Figure 7n and o), and q-PCR (Figure 7p), IL-6 expression was decreased by the RTX intervention compared to the CAP-injected group, which was similar to the trends of fibrosis and angiogenesis indices in the rabbit ear HS model.

To account for the high level of IL-6 expression in HS, we assessed IL-6 expression in HUVECs incubated with CAP. The western blotting (Figure 7q and r) and q-PCR (Figure 7s) results were consistent with the *in vivo* expression patterns observed (Figure 7g–k), demonstrating a significant increase in IL-6 expression in TRPV1-activated HUVECs. Increased IL-6 secretion was also detected in HUVECs treated with CAP (Figure 7t). The IL-6/STAT3 signaling is closely linked to angiogenesis and tumor progression, as IL-6 activates STAT3 to promote VEGF expression and downstream angiogenic processes [43, 44]. GSEA analysis also revealed significant positive enrichment of the IL-6/STAT3 pathway (Figure S5e and f, see online supplementary material). To verify whether the IL-6/STAT3 pathway plays a vital role in regulating angiogenesis under activation of the TRPV1 channel, we profiled the phosphorylation status at Tyr705 (pY705-STAT3) in CAP-treated HUVECs using western blotting analysis. As expected, the expression level of pY705-STAT3 increased with increasing CAP concentrations (Figure 7u and v). Furthermore, we examined pY705-STAT3 expression in rabbit ear HS tissues (Figure 7w–z), while these results confirmed that pY705-STAT3 was upregulated in the CAP-injected groups and downregulated in the RTX-ablation group. Taken together, our data provide strong evidence that IL-6 signaling promotes increased pY705-STAT3 activity upon TRPV1 channel activation, consequently enhancing angiogenesis.

### The TRPV1/NF-κB/IL-6 axis regulates angiogenesis in HUVECs via the STAT3 signaling pathway

**Figure 8:**
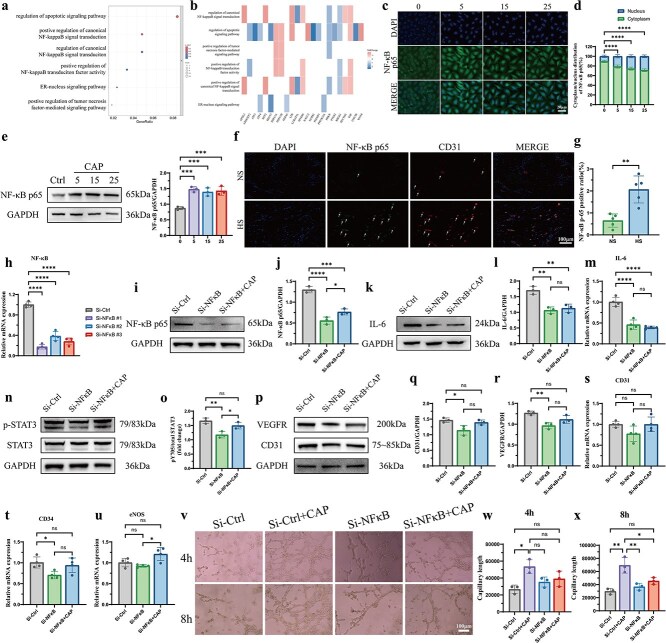
The TRPV1/NF-κB/IL-6 axis regulates the angiogenesis of HUVECs via the STAT3 signaling pathway. (**a**) Enrichment plots of GO terms related to NF-κB signaling between normal HUVECs (control group, *n* = 3 biologically independent samples) and HUVECs treated with the TRPV1 channel agonist CAP (treated group, *n* = 3 biologically independent samples). (**b**) Heatmap displaying the expression levels of genes associated with NF-κB signaling GO terms. Up- and downregulated genes are highlighted in red and blue, respectively. (**cd**) Representative immunostaining images (**c**) and quantitative analysis (**d**) of NF-κB p65 nuclear translocation in HUVECs treated with varying concentrations of CAP (0, 5, 15, and 25 μM). *n* = 3 independent experiments. Scale bar: 20 μm (**e**) NF-κB p65 expression levels in HUVECs treated with 0, 5, 15, and 25 μM CAP measured by western blotting. *n* = 3 independent experiments. (**fg**) Representative NF-κB p65 immunostaining images (**f**) and quantitative analysis (**g**) comparing NS samples and HS samples. Scale bar: 100 μm. *n* = 5 biologically independent samples. (**h**) The inhibitory efficiency of siRNAs targeting NF-κB was assessed using q-PCR. *n* = 4 independent experiments. (**ij**) NF-κB p65 expression levels in Si-NF-κB-transfected HUVECs, with or without CAP treatment, measured by western blotting. *n* = 3 independent experiments. (**kl**) IL-6 expression levels in Si-NF-κB-transfected HUVECs, with or without CAP treatment, measured by western blotting; *n* = 3 independent experiments. (**m**) IL-6 expression levels in Si-NF-κB-transfected HUVECs, with or without CAP treatment, measured by q-PCR. *n* = 4 independent experiments. (**no**) Representative blots and quantitative analysis of p-STAT3 over total STAT3 in Si-NF-κB-transfected HUVECs, with or without CAP treatment. *n* = 3 independent experiments. (**p**–**r**) VEGFR and CD31 expression levels in Si-NF-κB-transfected HUVECs, with or without CAP treatment, measured by western blotting. *n* = 3 independent experiments. (**s**–**u**) CD31 (**s**), CD34 (**t**), and eNOS (**u**) expression levels in Si-NF-κB-transfected HUVECs, with or without CAP treatment, measured by q-PCR. *n* = 4 independent experiments. (**v**–**x**) Representative optical images (**v**) of the *in vitro* tubular network formed by Si-NF-κB-transfected HUVECs, with or without CAP treatment; quantitative analysis of capillary length is shown at 4 (**w**) and 8 h (**x**). Scale bar: 100 μm. *n* = 3 independent experiments; data are presented as the mean ± SD. ns, not significant, ^*^P < 0.05, ^**^P < 0.01, ^***^P < 0.001, ^****^P < 0.0001. *HUVECs* human umbilical vein endothelial cells, *GO* gene ontology, *CAP* capsaicin, *NS* normal skin, *HS* hypertrophic scar, *SiRNA* small interfering RN, *si-NF-κB* NF-κB small interfering RNA, *si-Ctrl* negative control small interfering RNA, *q-PCR* quantitative real time PCR

Next, we performed a GO analysis for terms related to signaling pathways, which indicated that NF-κB signaling regulation was significantly altered following TRPV1 activation (Figure 8a). We then analyzed the genes that were highly expressed in the aforementioned signaling pathways using a heatmap, which indicated that these pathways were activated (Figure 8b). Simultaneously, we compared the signaling pathways enriched in the GO analysis with those in the KEGG analysis (Figure 7d) and found that only the NF-κB signal pathway was simultaneously enriched. Furthermore, we employed IF staining to verify that activation of TRPV1 promoted the nuclear translocation of NF-κB p65 in HUVECs (Figure 8c and d). Western blotting analysis results also showed that the expression of NF-κB p65 was upregulated in CAP-treated HUVECs (Figure 8e).

To confirm our observations in HUVECs, we performed IF staining on skin tissues derived from NS and HS. IF staining indicated that CD31 and NF-κB p65 were aggregated and highly expressed in HS compared to NS (Figure 8f and g). In addition, we examined the expression of NF-κB p65 in the rabbit ear HS tissues treated with CAP using western blotting and IF staining (Figure S6a–d, see online supplementary material). These results confirmed a significant increase in the NF-κB p65 expression. Finally, we assessed the NF-κB p65 expression in rabbit ear HS tissues following the ablation of the TRPV1 channel. Using western blotting and IF staining (Figure S6e–h, see online supplementary material), we observed that RTX intervention decreased the NF-κB p65 expression compared to the CAP-injected group, mirroring the trends of angiogenesis indices and IL-6 expression in the rabbit ear HS model.

Previous studies have shown that NF-κB is an important upstream gene of IL-6 and a key regulatory signal for angiogenesis [45]. We then examined whether the promotion of endothelial cell function by IL-6/STAT3 under TRPV1 activation is dependent on NF-κB signal transduction. We used siRNA to interfere with NF-κB expression in CAP-cultured HUVECs. The silencing efficiency of si-NF-κB#1 was the highest according to q-PCR analysis, and thus si-NF-κB#1 was selected for subsequent experiments (Figure 8h). Western blotting results confirmed that NF-κB expression could no longer be increased by CAP following NF-κB knockdown (Figure 8i and j). We then assessed potential downstream and angiogenesis-related genes in response to NF-κB interference. Western blotting and q-PCR results showed that the protein and mRNA expression level of IL-6 were decreased in HUVECs (Figure 8k–m), which was consistent with the reduced expression of NF-κB. In addition, TRPV1 activation did not significantly increase the expression level of pY705-STAT3 in the case of NF-κB knockdown (Figure 8n and o). We also assessed the protein expression of angiogenesis-related markers (CD31 and VEGFR), which showed a significant decrease after NF-κB knockdown and no longer exhibited a dominant increase when treated with CAP in HUVECs (Figure 8p–r). q-PCR analysis showed similar trends, indicating that the mRNA expression of CD31, CD34, and eNOS was not statistically significantly increased following TRPV1 activation when NF-κB was knocked down (Figure 8s–u). Additionally, we performed Matrigel tube-formation assay which demonstrated a significantly reduced angiogenesis capacity of Si-NF-κB-interfered HUVECs, based on tube capillary length (Figure 8v–x).

In addition to siRNA-mediated NF-κB knockdown, we investigated the pharmacological NF-κB inhibitor BAY11-7082, which showed that inhibition of NF-κB expression significantly suppressed the protein and mRNA expression of IL-6 (Figure S7a–c, see online supplementary material). Meanwhile, TRPV1 activation did not significantly increase the phosphorylation level of pY705-STAT3 under pharmacological NF-κB inhibition (Figure S7d and e, see online supplementary material). Next, we assessed the protein and mRNA expression of angiogenesis-related markers (CD31 and VEGFR), which demonstrated a significant decrease after pharmacological NF-κB inhibition and no longer exhibited a dominant increase when treated with CAP in HUVECs (Figure S7f–j, see online supplementary material).

Furthermore, we performed rescue experiments in which rhIL-6 was supplemented following siRNA-mediated NF-κB knockdown. The protein levels of angiogenesis-related markers (CD31 and VEGFR) were significant increased after rhIL-6 treatment (Figure S8a–c, see online supplementary material). q-PCR analysis showed similar trends, indicating that the mRNA expression of CD31 and CD34 significantly increased following rhIL-6 treatment when NF-κB was knocked down (Figure S8d and e, see online supplementary material). Finally, we found that rhIL-6 treatment significantly enhanced the angiogenesis capacity of Si-NF-κB-interfered HUVECs, as evidenced by the number of branch points and tube length in the Matrigel tube-formation assay (Figure S8f–j, see online supplementary material). Notably, incomplete restoration of the angiogenic phenotype indicates involvement of additional downstream effectors other than the TRPV1/NF-κB/IL-6 axis, which is consistent with the pleiotropic nature of NF-κB signaling [26]. Our data demonstrate that IL-6/STAT3 represents a major downstream effector contributing to TRPV1-mediated angiogenic responses, while additional NF-κB-dependent pathways are likely to contribute to the residual angiogenic activity. Overall, these results suggest that CAP upregulates transcription factor NF-κB and the major downstream target IL-6/STAT3 to promote angiogenesis primarily via TRPV1 activation in HUVECs.

### Increased expression of the TRPV1/NF-κB/IL-6 axis in HS is positively correlated with scar angiogenesis and may indicate poor prognosis

**Figure 9:**
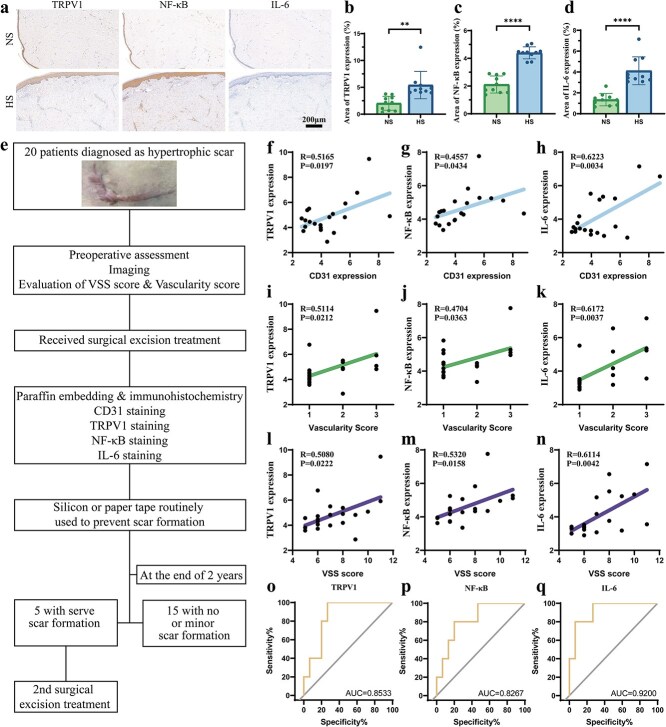
Increased expression of the TRPV1/NF-κB/IL-6 axis in HS is positively correlated with scar angiogenesis and may indicate poor prognosis. (**a**–**d**) Representative immunostaining images for TRPV1, NF-κB, and IL-6 (a) and quantitative analysis (b–d) comparing NS samples (*n* = 10 biologically independent samples) and HS samples (*n* = 10 biologically independent samples). (**e**) Schematic flow of patient admission and follow-up for those diagnosed with HS disease. (**f**–**h**) Correlations analyses of CD31 IHC expression with TRPV1 (**f**), NF-κB (**g**), and IL-6 (**h**) IHC expression using Pearson’s correlation coefficient in HS patients. *n* = 20 biologically independent samples. (**i**–**k**) Correlation analyses of the vascularity score with TRPV1 (**i**), NF-κB (**j**), and IL-6 (**k**) IHC expression using Pearson’s correlation coefficient in HS patients. *n* = 20 biologically independent samples. (**l**–**n**) Correlation analyses of the VSS score with TRPV1 (**l**), NF-κB (**m**), and IL-6 (**n**) IHC expression using Pearson’s correlation coefficient in HS patients. *n* = 20 biologically independent samples. (**o**–**q**) ROC curve results for TRPV1 (**o**), NF-κB (**p**), and IL-6 (**q**) IHC expression in predicting poor prognosis for HS disease. *n* = 20 biologically independent samples. An AUC close to 1 indicates better diagnostic accuracy, with AUC values of 0.5–0.7 indicating low accuracy, 0.7–0.9 indicating certain accuracy, and >0.9 indicating high accuracy; data are presented as the mean ± SD. ns, not significant. ^**^P < 0.01, ^****^P < 0.0001. *HS* hypertrophic scar, *NS* normal skin, *IHC* immunohistochemistry, *VSS* Vancouver Scar Scale, *ROC* receiver operating characteristic, *AUC* area under the curve

Based on the results regarding the expression of key genes in HUVECs and rabbit HS tissues, we compared the expression of the TRPV1/NF-κB/IL-6 axis in the dermis of skin from HS patients with that in NS tissue using immunohistochemistry (Figure 9a). The results were consistent with the expression patterns observed in rabbit HS tissues and HUVECs (Figure 9b–d), showing a significant increase in TRPV1/NF-κB/IL-6 expression. To investigate TRPV1/NF-κB/IL-6 axis-dependent alterations in angiogenesis and the prognosis of human HS, specimens were obtained from 20 patients diagnosed with HS and received surgical excision treatment (Figure 9e). To further assess the contribution of increased expression of TRPV1, NF-κB, and IL-6 to angiogenesis in HS, we analyzed the correlations between CD31 expression, vascularity score, and the expression levels of TRPV1, NF-κB, and IL-6. We found that while all three markers showed positive correlations with CD31 expression (Figure 9f–h), the correlation was strongest for TRPV1 and IL-6, with NF-κB displaying a weaker positive correlation. Regarding the vascularity score, IL-6 exhibited a robust positive correlation (Figure 9i–k). Furthermore, we investigated the relationship between these markers and the VSS score (an indicator of scar severity). The results revealed that TRPV1, NF-κB, and IL-6 were all significantly and positively correlated with the VSS score (Figure 9l–n). Finally, we investigated whether the expression of TRPV1/NF-κB/IL-6 axis correlates with poor prognosis in our 20 HS patients by using ROC curves. The AUC result for IL-6 reached 0.9200 in the training set (Figure 9q), demonstrating its superior predictive power compared to TRPV1 (AUC = 0.8533; Figure 9o) and NF-κB (AUC = 0.8267; Figure 9p). These results collectively indicate that the expression levels of the TRPV1/NF-κB/IL-6 axis serve as robust indicators for predicting poor prognosis in HS disease.

## Discussion

Typical pathological scars result from excessive cutaneous wound healing and are considered prototypical of skin fibrosis, characterized by high treatment resistance; they are often regarded as benign skin tumors [46]. Although the fibrotic and inflammatory phenotypes (including erythematous, pruritic, painful, raised, or contractile qualities) vary among individuals, heightened vascularized redness at the scar site strongly indicates increased vascular density and hyperactive function [5]. Consistent with this phenomenon, based on scRNA-seq data, we identified an increased proportion of vascular endothelial cells and associated factors in our HS samples, with multiple angiogenesis-related enrichments compared with those in the NS samples. Previous studies have shown that one of the important reasons for tumor proliferation is the disruption of the “angiogenic switch” balance [47]. HS is considered as benign tumor disease, yet it also demonstrates pathological angiogenesis and micro-vessel proliferation, with the results of immunohistochemical staining further supporting this observation. These findings suggest that, although fibroblasts play a dominant role, vascular endothelial cells and the upregulated, uncontrolled angiogenic cascade are essential for pathological scar formation [48].

Notably, both clinical and anecdotal evidence has consistently demonstrated that improper long-term lifestyle choices, including spicy diet, exposure to sunlight and hot baths, may lead to exacerbation of the sickness and the development of symptoms, ultimately followed by scar formation and recurrence [5]. Nonetheless, the underlying mechanism responsible for this phenomenon remains poorly understood. As mentioned earlier, these lifestyle habits may lead to the opening of the TRPV1 channel; therefore, we believe that the activation of TRPV1 is closely related to the formation of HS. In particular, previous studies have confirmed that the TRPV1 channel plays a critical role in endothelial cells. For example, TRPV1-mediated calcium influx can activate PKA and protein kinase G, thereby regulating vascular tone [49]. Although most studies on TRPV1 have focused on its role in cardiovascular and neurological diseases [50, 51], our study reveals that the neurokinin-1 receptor, a downstream receptor related to TRPV1, participates in skin wound healing [52]. Based on the aforementioned research background and experimental results from clinical specimens, we hypothesized that TRPV1 activation stimulates local endothelial cell angiogenesis in scars, promoting scar hyperplasia, and we verified this hypothesis using a rabbit ear scar model and *in vitro* cell experiments. Therefore, we advocate that patients with HS adopt lifestyle modifications that reduce TRPV1 activation, including a bland diet, lukewarm water bathing, and minimized UV exposure. Besides, our findings suggest that therapeutic approaches for severe HS could explore local modulation of TRPV1 as a potential treatment strategy.

While circulating CAP levels in human plasma after dietary consumption are generally in the nanomolar range, several studies have reported that local tissue concentrations, particularly in the oral and gastrointestinal mucosa or skin following topical application, can be much higher [53–55]. In our *in vitro* experiments, we used 5 μM CAP to stimulate HUVECs for transcriptomic analysis, a concentration that is sufficient to induce TRPV1-mediated angiogenic responses characteristic of scar formation, and the results suggested that IL-6 was emphasized as the most likely candidate for mediating the TRPV1-regulated angiogenesis process in HS. As a proinflammatory cytokine known to play a key role in mediating inflammation-induced angiogenesis, numerous studies have revealed that IL-6 is an important regulator in cancer, cerebral, cardiovascular, and retinal diseases [56, 57]. IL-6 exerts many of its functions via the activation and phosphorylation of its major downstream effector, the transcription factor STAT3 [58]. Our further investigation confirmed the overexpression of IL-6 in human HS samples and verified that TRPV1 contributes to the amplified IL-6/STAT3 signaling both *in vitro* and *in vivo*. Given that several existing studies have reported the IL-6 and STAT3 signaling pathways in HS, our current study is the first to focus on HUVECs and their angiogenic functions [59]. Moreover, although IL-6 was previously considered to be produced mainly by T cells [60], our current results demonstrate that TRPV1 stimulation enhances IL-6 secretion by HUVECs. IL-6 can also regulate the differentiation and survival of T cells, thus perpetuating chronic inflammation and ensuring the continuous production of the growth factors and cytokines required for ECM deposition and angiogenesis [61, 62]. In this way, long-term TRPV1 activation persistently sustains inflammation-associated angiogenesis, fueling scar initiation, progression, and proliferation. Interestingly, the level of IL-6 and STAT3 activation through phosphorylation at Tyr705 was mostly observed, but not completely reversed upon TRPV1 blockade. Simultaneously, the *in vivo* expression of VEGFA and CD31 was significantly increased by TRPV1 stimulation in a concentration-dependent manner, which is believed to serve as paracrine proangiogenic cytokines that modulate adjacent fibroblasts in turn [63]. Here, we demonstrate that TRPV1 stimulation caused by long-term spicy diet can enhance angiogenesis in HS, mainly through a direct modulation of VECs. These data sufficiently link the IL-6/STAT3 cascade to TRPV1 function and establish IL-6/STAT3 as a dominant downstream target of TRPV1-mediated activation in HUVECs to modulate scar formation.

Although the production of IL-6 is regulated by various factors, one of the most classic mechanisms is the induction of IL-6 mRNA expression via activation of the NF-κB signaling pathway [64]. Activation of the NF-κB signaling pathway has been confirmed to be associated with Ca^2+^, and transcriptome sequencing results suggest that CAP can activate this pathway in HUVECs. Subsequently, we demonstrated that TRPV1 promotes IL-6 secretion and regulates angiogenesis by activating NF-κB signaling pathway through *in vitro* knockdown and replenishment experiments, and NF-κB is broadly recognized for its importance in cellular biological functions [45]. The central paradigm of NF-κB regulation involves the translocation of active NF-κB from the cytoplasm to the nucleus, while the posttranslational modification of p65 is critical for regulating its transcriptional activity [65]. The activation of NF-κB enhances inflammation by increasing the expression of downstream pro-inflammatory cytokines, including IL-6 and tumor necrosis factor alpha (TNF-α), which in turn stimulate tissue proliferation [66]. Previous studies have shown that NF-κB activation aggravates pathological scarring by mediating TRPC3 or Notch1 expression [67, 68], and our results in HUVECs suggest that the IL-6/STAT3 pathway is a critical NF-κB-dependent pro-angiogenic signaling mechanism in HS, and is mediated by the upstream TRPV1 receptor. Additionally, NF-κB and STAT3 may act in positive feedback loops to enhance the production of IL-6, CD31 and VEGF, which recruit additional immune cells that sustain chronic inflammation, angiogenesis, and fibrosis [69]. Notably, NF-κB is known to regulate multiple pro-angiogenic mediators beyond IL-6, including TNF-α, IL-8, and myelocytomatosis oncogene (MYC), each of which can independently promote endothelial survival, proliferation, or vascular remodeling [70–72]. Herein, we do not propose exclusivity of the TRPV1/NF-κB/IL-6 axis, but rather identify it as an important and experimentally validated signaling route mediating TRPV1-driven angiogenesis in endothelial cells. The incomplete rescue by IL-6 underscores the necessity of considering the broader crosstalk network for comprehensive therapeutic strategies.

Clinical and basic research has shown that early intervention in scars after wound healing can shorten the immature stage, improve the ultimate outcome, and effectively prevent and control the occurrence and development of pathological scars [6]. However, pathological scars arise from multiple factors, and the specificity of early intervention needs further improvement. Increasing evidence suggests that, despite the lack of specific biomarkers, there are relevant molecules that can provide clues in the early stages of scar formation and wound healing [73]. For instance, our team has identified the NKG2A-soluble soluble human leukocyte antigen E (HLA-E) axis as a predictive biomarker and potential therapeutic target [74]. This study demonstrated that activation of the TRPV1/NF-κB/IL-6 axis in endothelial cells can promote angiogenesis and induce HS formation. Additionally, we examined the relationships between the expression of TRPV1, NF-κB, IL-6 and prognosis, finding that overexpression of these factors was strongly correlated with severe symptoms or recurrence of HS among 20 consecutive HS patients. Therefore, we believe that the TRPV1/NF-κB/IL-6 axis can aid in the early diagnosis and prognosis of HS.

Abnormal vessel growth and function are hallmarks of cancer and inflammatory diseases, where endothelial cells significantly contribute to disease progression, despite the involvement of various cell types [75]. Beneficial angiogenesis primarily occurs in specific physiological processes, such as wound healing and the reproductive cycle [76]. Thus, regulating pathological angiogenesis is a relatively safe treatment strategy for diseases [77]. The role of TRPV1 in angiogenesis during tumor development has garnered attention as a potential therapeutic target [78]. In recent years, inhibiting angiogenesis has also been explored as a strategy for treating HS [79]. As mentioned, we have established a close relationship between TRPV1, downstream molecules, and angiogenesis in HS, providing new targets and concepts for the treatment of HS in the future.

## Conclusions

Overall, our findings offer a comprehensive understanding of how TRPV1-mediated angiogenesis induces HS formation and proliferation through the amplification of NF-κB/IL-6 signaling. Several limitations of the present study warrant mention to guide future research. First, the cohort size remains limited due to the scarcity of surgically excised HS specimens, although we expanded our analysis by integrating an independent public scRNA-seq dataset (GSE156326). Despite this limitation, the analysis successfully identified novel molecular mechanisms underlying the observed phenotype and these novel mechanisms warrant validation in substantially larger, independent cohorts to confirm their biological significance and generalizability. Second, HUVECs exhibited compromised characteristics when compared to primary dermal microvascular endothelial cells. Third, since cellular crosstalk within HS microenvironments remains unclear, further research would benefit from applying the endothelial cell-specific TRPV1 knockout model. Lastly, due to variations in species, metabolic pathways, pharmacokinetics, and distribution *in vivo*, the gap between dietary CAP exposure and systemic administration remains obscure. Further insights from chronic low-dose dietary studies would be beneficial. Altogether, our findings suggest that TRPV1 and its downstream effectors may serve as novel diagnostic, prognostic, and therapeutic targets for HS, warranting further clinical trials to evaluate their usefulness and significance.

## Supplementary Material

tkag009_NSupporting_information

## Data Availability

All data needed to evaluate the conclusions in the paper are present in the paper and/or the Supplementary Materials.
