# Supplementary material for: Modulating hypertrophic scar formation by targeting endothelial transient receptor potential vanilloid-1/nuclear factor kappa-B/interleukin-6 axis to regulate angiogenesis
Source: Burns Trauma. 2026 Jan 16;14:tkag009. doi: 10.1093/burnst/tkag009 (PMC13162238; doi:10.1093/burnst/tkag009)
Supplement: tkag009_NSupporting_information [file tkag009_nsupporting_information.docx]

Supporting Information for

**Modulating Hypertrophic Scar Formation by Targeting Endothelial TRPV1/NF-κB/IL6 Axis to Regulate Angiogenesis**

Hao Ma *et al.*

*Corresponding author. Email: aru_ren@msn.com (P.M.)

**This PDF file includes:**

Supplementary Text

Figs. S1 to S8

Tables S1 to S5

Supplementary Text

Fig.S1.


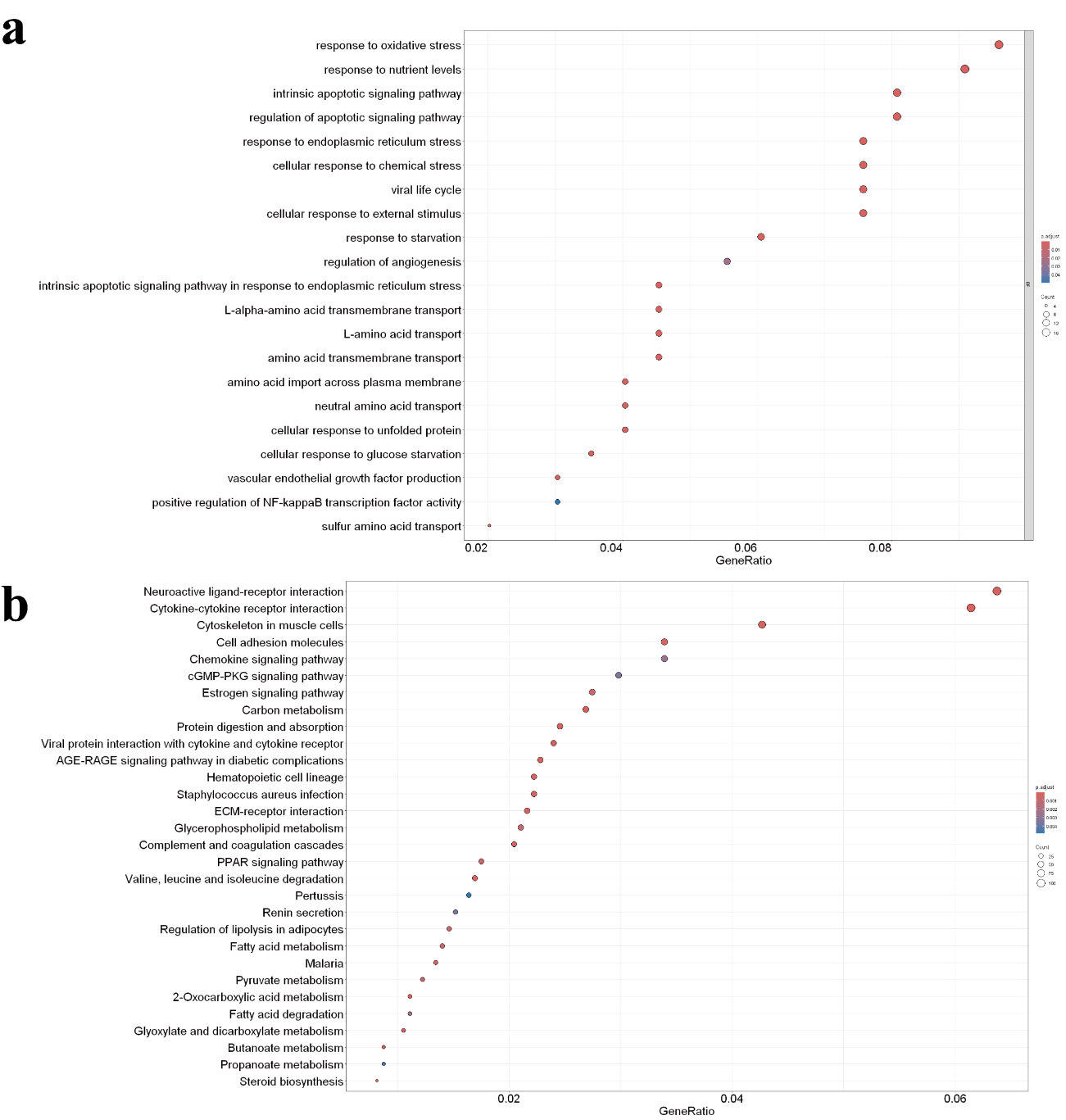


**GO and KEGG pathway analysis of Bulk RNA sequencing between** **three NS samples and three HS samples.** (**a**) Enrichment plots of GO terms based on the DEGs. (**b**) Enrichment plots of KEGG pathways using the DEGs. *GO* Gene Ontology, *KEGG* Kyoto Encyclopedia of Genes, *NS* normal skin, *HS* hypertrophic scar, *DEGs* differentially expressed genes

Fig.S2.


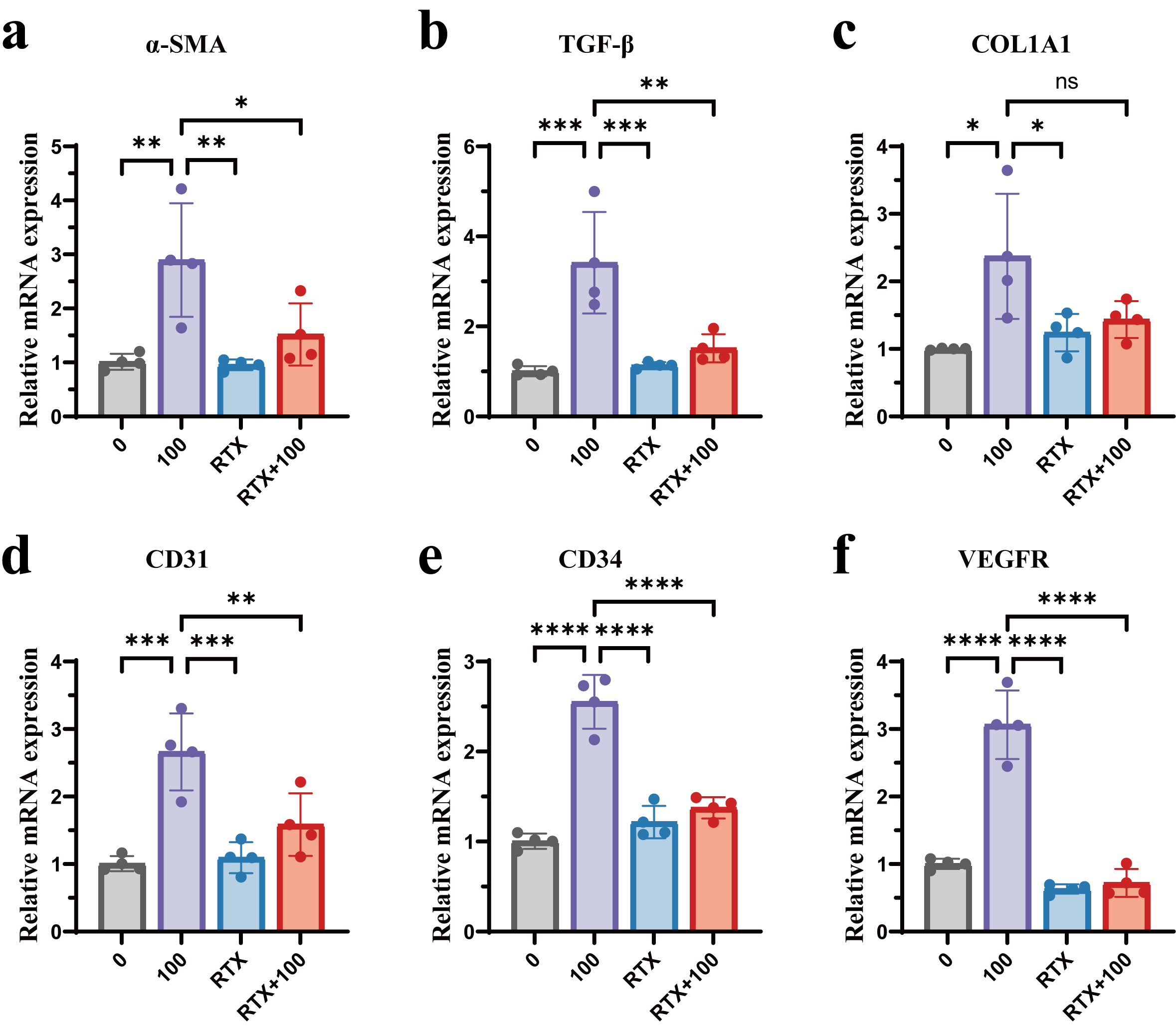


**mRNA expression levels of fibrosis and angiogenesis markers in hypertrophic scars.** (**a**-**c**) Expression levels of α-SMA (a), TGF-β (b), and COL1A1 (c) in hypertrophic scars of the four groups, measured by q-PCR. Four groups were divided: no treatment (0), 100 mg/kg CAP injected (100), RTX pre-treated, RTX pre-treated with 100 mg/kg CAP injected (RTX+100). n=4 biologically independent samples. (**d**-**f**) Expression levels of CD31 (d), CD34 (e), and VEGFR (f) in hypertrophic scars of the four groups, measured by q-PCR. n=4 biologically independent samples. Data are presented as the Mean±SD. ns, not significant, * P<0.05, ** P<0.01, *** P<0.001, **** P<0.0001. *CAP* capsaicin, *RTX* resiniferatoxin, *q-PCR* Quantitative real time PCR

Fig.S3.


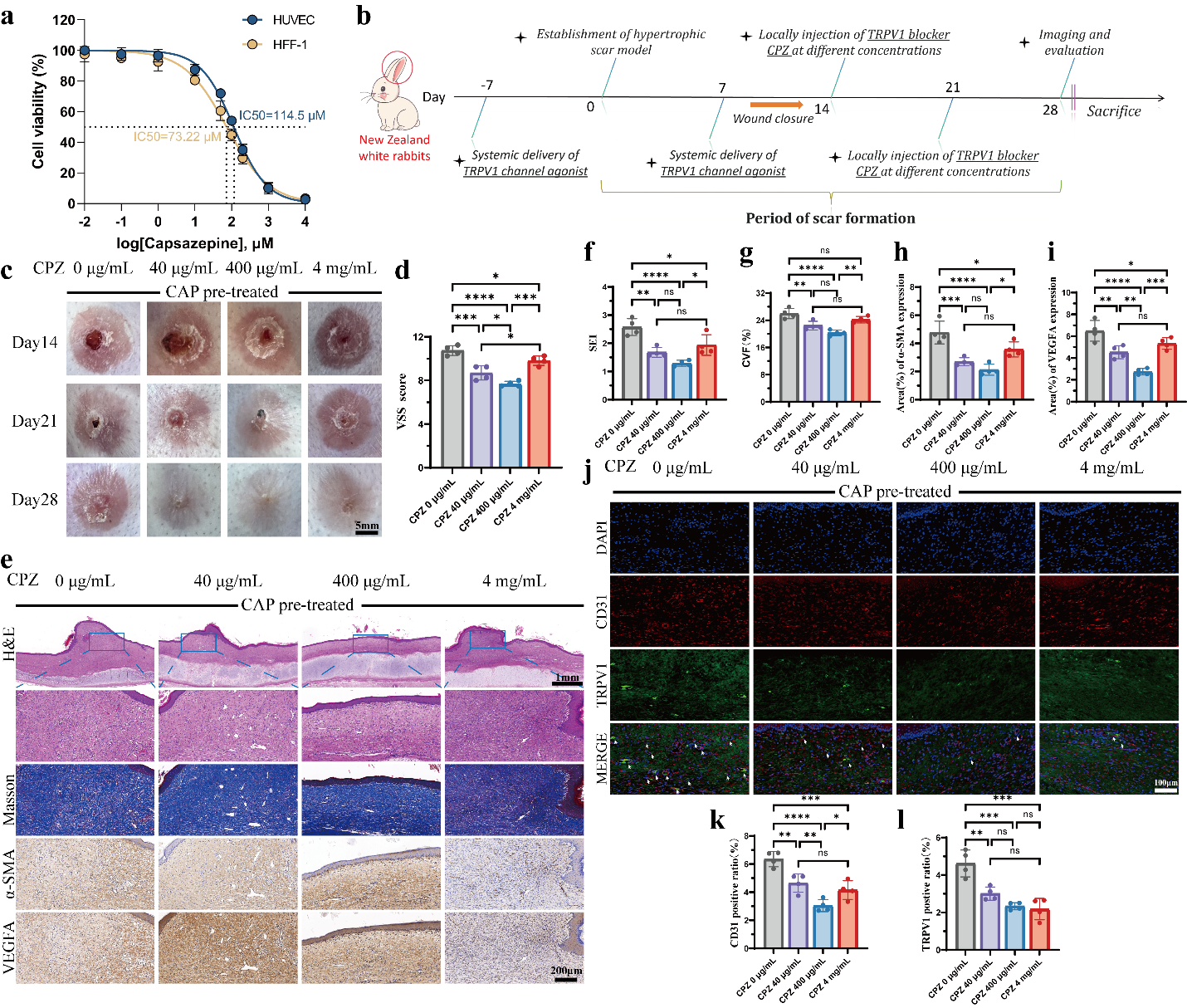


**Local injection of the TRPV1 blocker CPZ alleviates scar fibrosis and angiogenesis induced by CAP in vivo.** (**a**) Cell viability of HUVECs and HFF-1 were assessed using the CCK8 assay with different concentrations of the TRPV1 channel blocker capsazepine after incubation for 24 hours. n=5 independent experiments. (**b**) Schematic workflow of the rabbit ear HS model establishment and evaluation. Four groups were all pre-treated with CAP and then injected with 0 μg/mL, 40 μg/mL, 400 μg/mL, 4 mg/mL CPZ. n=4 biologically independent samples. (**c**) Representative photographs of rabbit ear wounds with different treatments at day 14, 21, and 28. (**d**) VSS scores of the HS tissues in four groups at day 28. n=4 biologically independent samples. (**e**) Representative images of H&E staining, Masson staining, α-SMA immunostaining, and VEGFA immunostaining of HS tissues in the four groups at day 28. (**f**, **g**) Quantitative analysis of SEI (f) and CVF (g) of HS tissues in the four groups at day 28. n=4 biologically independent samples. (**h**, **i**) Quantitative analysis of the percentage of α-SMA-positive areas (h) and VEGFA-positive areas (i) in the four groups at day 28. n=4 biologically independent samples. (**j**-**l**) Representative coimmunostaining images for CD31 and TRPV1 (j) with quantification of the CD31 (k) and TRPV1 (l) positive ratios in the four groups. n=4 biologically independent samples. Data are presented as the Mean±SD. ns, not significant, * P<0.05, ** P<0.01, *** P<0.001, **** P<0.0001. *CPZ* capsazepine, *CAP* capsaicin, *HUVECs* human umbilical vein endothelial cells, *HFF-1* Human Foreskin Fibroblast-1, *CCK8* Cell Counting Kit 8, *HS* hypertrophic scar, *VSS* Vancouver Scar Scale, *SEI* scar evaluation index, *CVF* collagen volume fraction

Fig.S4.


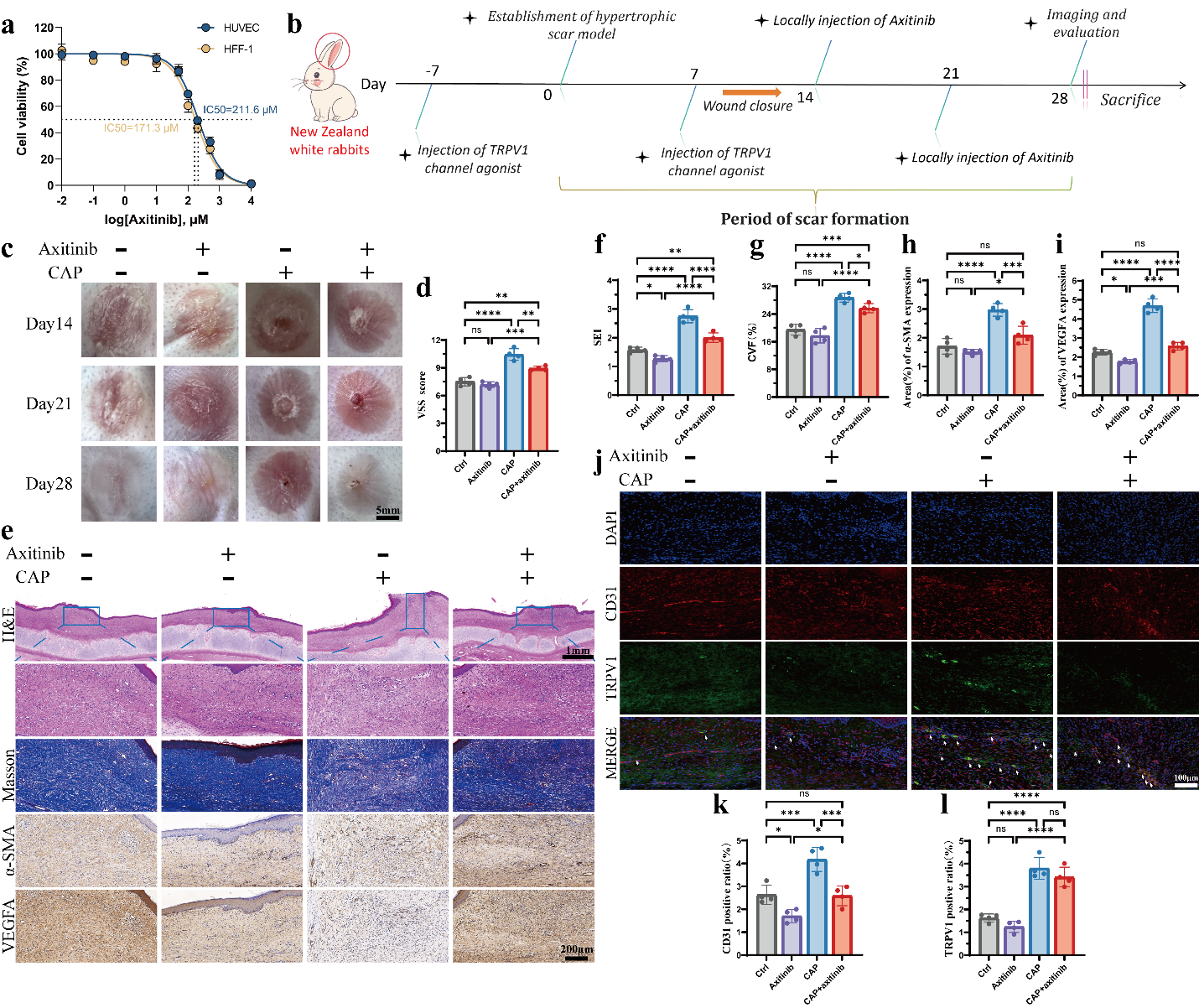


**Anti-angiogenic drugs Axitinib could alleviate scar formation induced by CAP in vivo.** (**a**) Cell viability of HUVECs and HFF-1 were assessed using the CCK8 assay with different concentrations of Axitinib after incubation for 24 hours. n=5 independent experiments. (**b**) Schematic workflow of the rabbit ear HS model establishment and evaluation. Four groups were divided: no treatment (Ctrl), 1.25 mg/mL Axitinib locally injected (Axitinib), 100 mg/kg pre-treated (CAP), 100 mg/kg CAP pre-treated with 1.25 mg/mL Axitinib locally injected (CAP+Axitinib). n=4 biologically independent samples. (**c**) Representative photographs of rabbit ear wounds with different treatments at day 14, 21, and 28. (**d**) VSS scores of the HS tissues in the 0, 100, RTX, and RTX+100 groups at day 28. n=4 biologically independent samples. (**e**) Representative images of H&E staining, Masson staining, α-SMA immunostaining, and VEGFA immunostaining of HS tissues in the four groups at day 28. (**f**, **g**) Quantitative analysis of SEI (f) and CVF (g) of HS tissues in the four groups at day 28. n=4 biologically independent samples. (**h**, **i**) Quantitative analysis of the percentage of α-SMA-positive areas (h) and VEGFA-positive areas (i) in the four groups at day 28. n=4 biologically independent samples. (**j**-**l**) Representative coimmunostaining images for CD31 and TRPV1 (j) with quantification of the CD31 (k) and TRPV1 (l) positive ratios in the four groups. n=4 biologically independent samples. Data are presented as the Mean±SD. ns, not significant, * P<0.05, ** P<0.01, *** P<0.001, **** P<0.0001. *CAP* capsaicin, *HUVECs* human umbilical vein endothelial cells, *HFF-1* Human Foreskin Fibroblast-1, *CCK8* Cell Counting Kit 8, *HS* hypertrophic scar, *VSS* Vancouver Scar Scale, *SEI* scar evaluation index, *CVF* collagen volume fraction

Fig.S5.


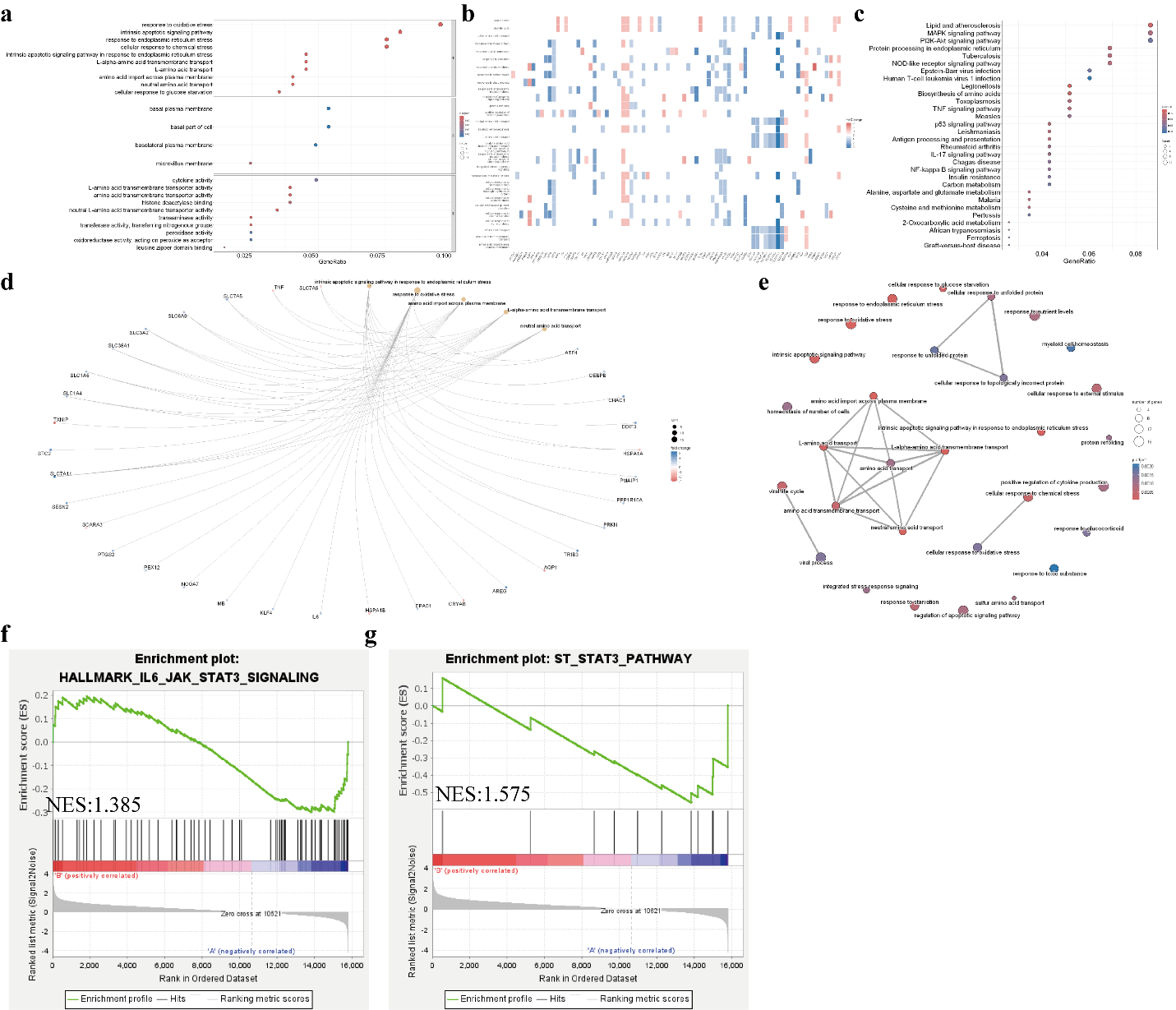


**Supplementary results of RNA transcriptome sequencing comparing normal HUVECs with HUVECs treated with the TRPV1 channel agonist capsaicin.** (**a**) Enrichment plots of GO terms between the control and treated group using the DEGs. (**b**) Heatmap displaying the expression levels of genes related to GO terms between the control and treated group, with color coding for upregulated (red) and downregulated (blue) genes. (**c**) Enrichment plots of KEGG pathways between the control and treated group using DEGs. (**d**) Enrichment interaction network of GO terms. (**e**) PPI Network of top 5 GO terms and related genes. (**f**, **g**) GSEA enrichment plots showing significant activation of the IL-6/STAT3 pathway in HUVECs treated with capsaicin (Group B) compared to normal HUVECs (Group A) (p-adj < 0.05, FDR < 0.25). *HUVECs* human umbilical vein endothelial cells, *GO* Gene Ontology, *DEGs* differentially expressed genes, *KEGG* Kyoto Encyclopedia of Genes, *PPI* Protein-Protein Interaction, *GSEA* Gene-set Enrichment Analysis, *p-adj* adjusted p-value, *FDR* False discovery rate, *NES* normalized enrichment score

Fig.S6.


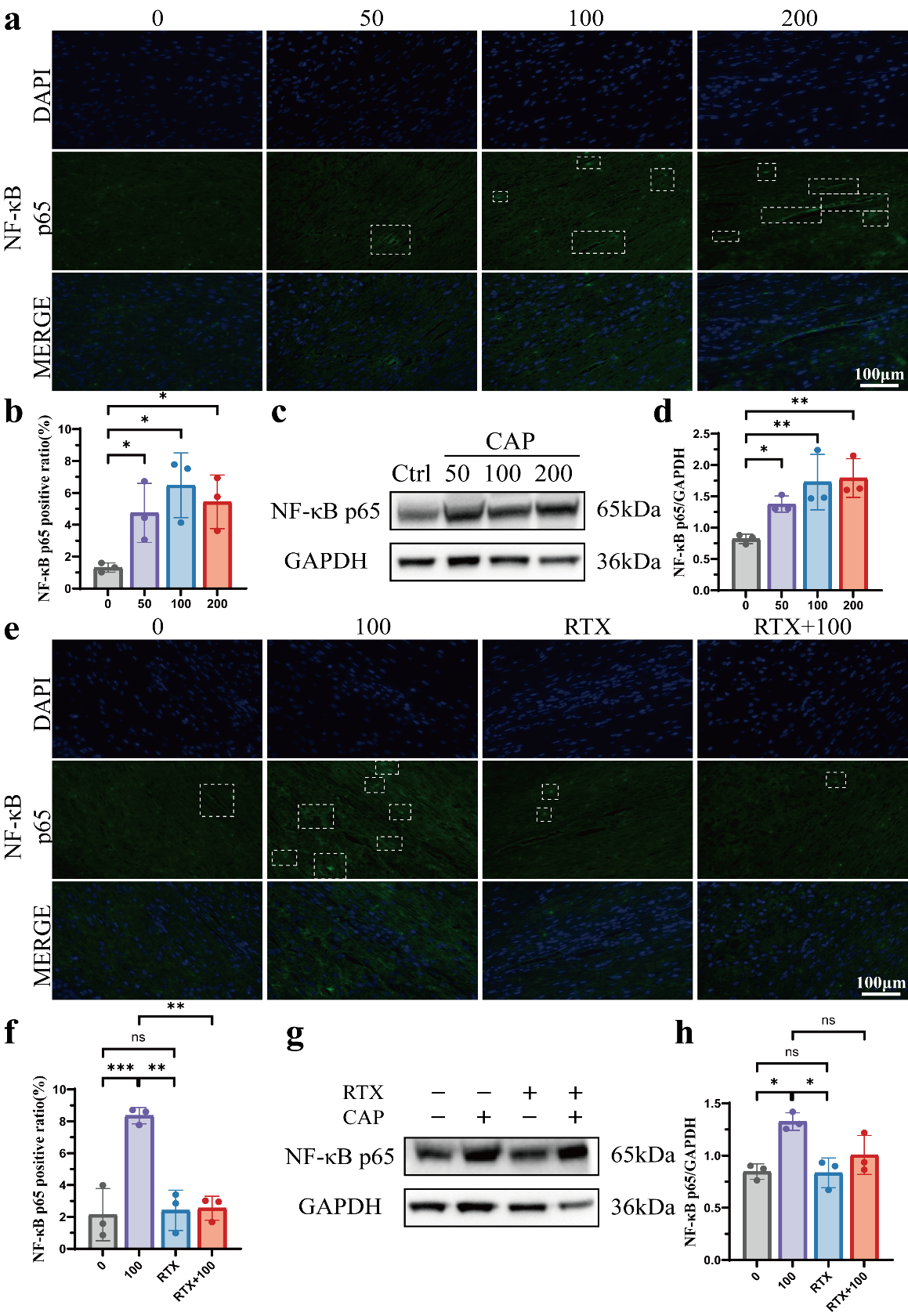


**The expression level of NF-κB p65 in the rabbit ear hypertrophic scars.** (**a**, **b**) Representative NF-κB p65 immunostaining images (a) and quantitative analysis (b) of hypertrophic scars across the 0. 50, 100, and 200 mg/kg groups at day 28. n=3 biologically independent samples. (**c**, **d**) NF-κB p65 expression levels in rabbit ear hypertrophic scars from groups treated with 0, 50, 100, and 200 mg/kg capsaicin at day 28, measured by western blotting. n=3 biologically independent samples. (**e**, **f**) Representative NF-κB p65 immunostaining images (e) and quantitative analysis (f) of hypertrophic scars in the 0. 100, RTX, and RTX+100 groups (0: no treatment, 100: 100 mg/kg CAP injected, RTX: RTX pre-treated, RTX+100: RTX pre-treated with 100 mg/kg CAP injected.) at day 28. n=3 biologically independent samples. (**g**, **h**) NF-κB p65 expression levels in rabbit ear hypertrophic scars in the 0. 100, RTX, and RTX+100 groups at day 28, measured by western blotting. n=3 biologically independent samples. Data are presented as the Mean±SD. ns, not significant, * P<0.05, ** P<0.01, *** P<0.001. *CAP* capsaicin, *RTX* resiniferatoxin

Fig.S7.


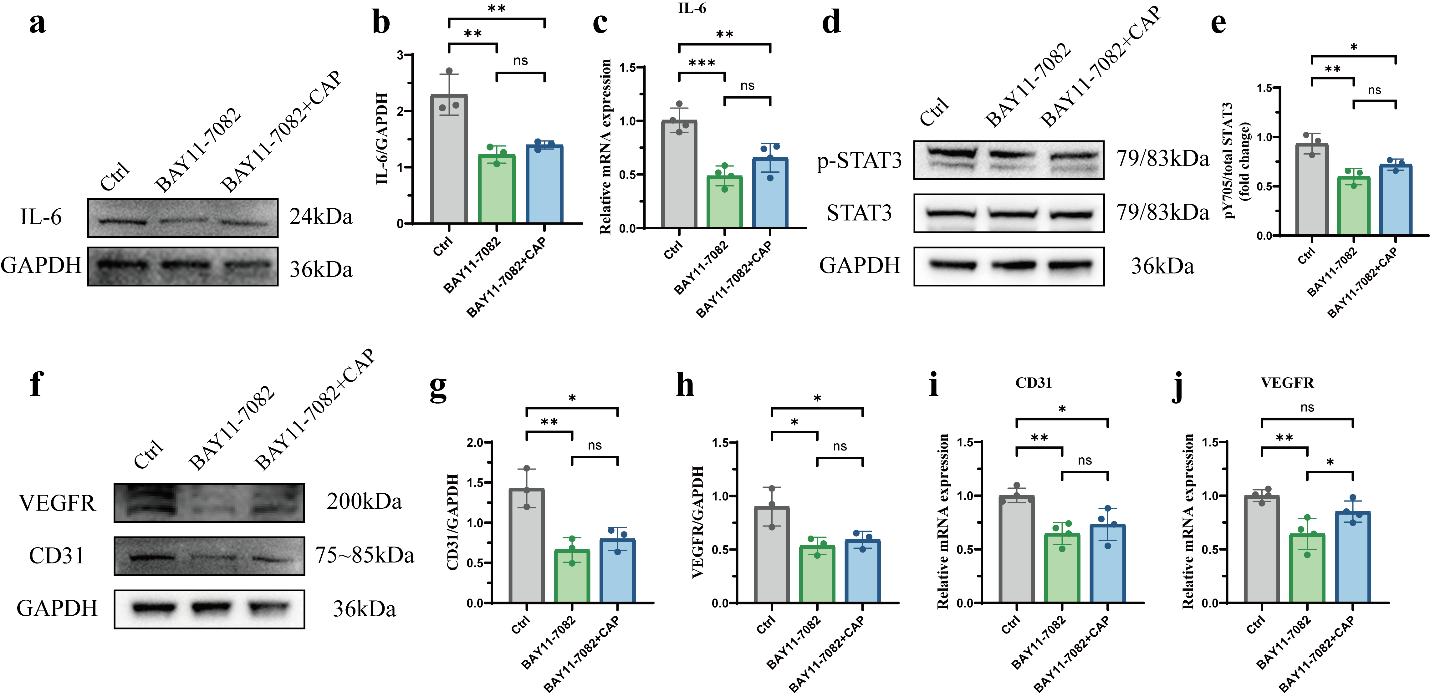


**Pharmacological NF-κB inhibition in HUVECs.** (**a**, **b**) IL-6 expression levels in BAY11-7082 pre-treated HUVECs, with or without followed capsaicin treatment, measured by western blotting. n=3 independent experiments. (**c**) IL-6 expression levels in BAY11-7082 pre-treated HUVECs, with or without followed CAP treatment, measured by q-PCR. n=4 independent experiments. (**d**, **e**) Representative blots and quantitative analysis of p-STAT3 over total STAT3 in BAY11-7082 pre-treated HUVECs, with or without followed capsaicin treatment. n=3 independent experiments. (**f**-**h**) VEGFR and CD31 expression levels in BAY11-7082 pre-treated HUVECs, with or without followed CAP treatment, measured by western blotting. n=3 independent experiments. (**i**, **j**) CD31 and VEGFR expression levels in BAY11-7082 pre-treated HUVECs, with or without followed capsaicin treatment, measured by q-PCR. n=4 independent experiments. Data are presented as the Mean±SD. ns, not significant, * P<0.05, ** P<0.01, *** P<0.001. *HUVECs* human umbilical vein endothelial cells, CAP capsaicin, *q-PCR* Quantitative real time PCR

**Fig.S8.**


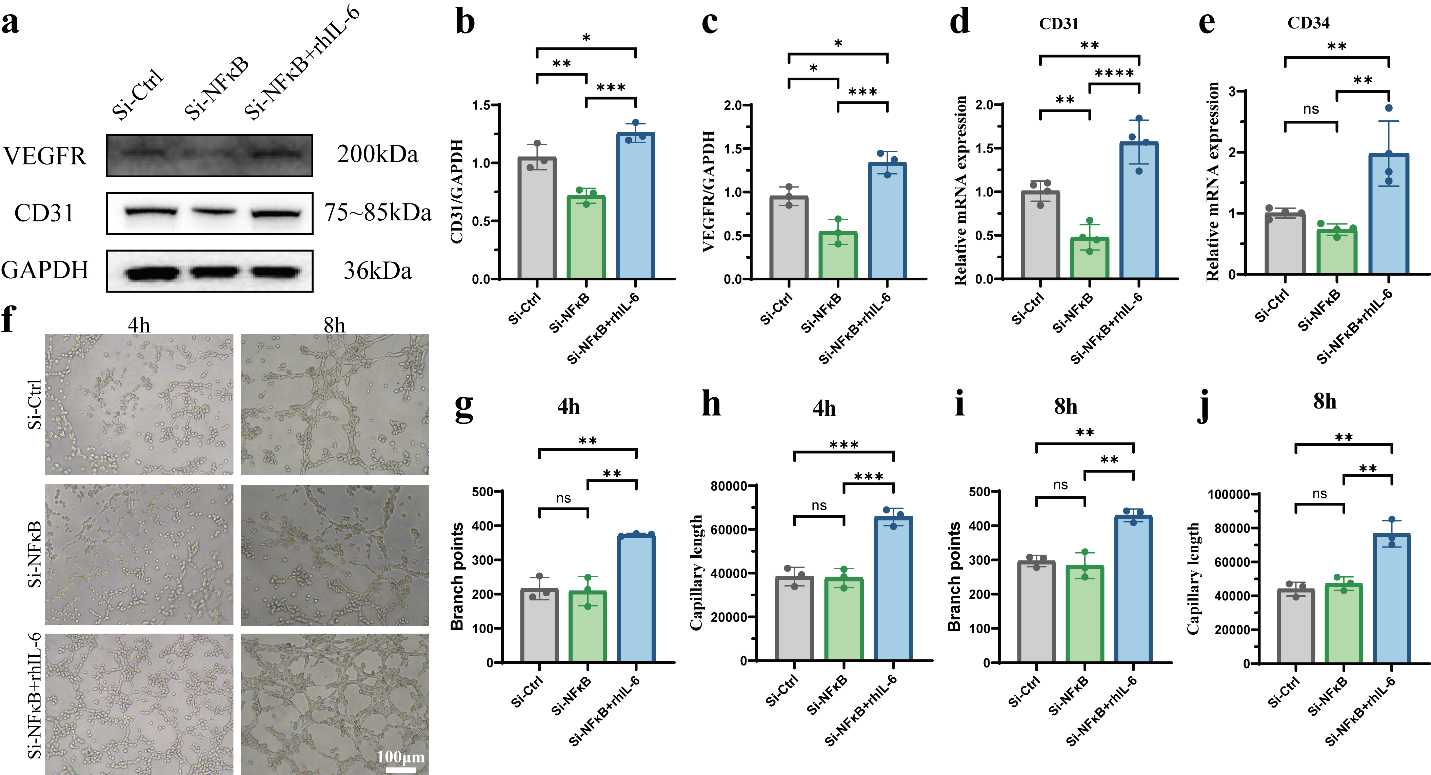


**Rescue experiments by supplementing rhIL-6 treatment.** (**a**-**c**) VEGFR and CD31 expression levels in Si-NFκB-transfected HUVECs, with or without rhIL-6 treatment, measured by western blotting. n=3 independent experiments. (**d**, **e**) CD31 and CD34 expression levels in Si-NFκB-transfected HUVECs, with or without rhIL-6 treatment, measured by q-PCR. n=4 independent experiments. (**f**-**j**) Representative optical images (f) of the in vitro tubular network formed by Si-NFκB-transfected HUVECs, with or without rhIL-6 treatment. Quantitative analyses of branch points and capillary length are shown at 4 hours (g, h) and 8 hours (i, j). n=3 independent experiments. Data are presented as the Mean±SD. ns, not significant, * P<0.05, ** P<0.01, *** P<0.001, **** P<0.0001. *rhIL-6* recombinant human IL-6, *si-Ctrl* negative control small interfering RNA, *si-NFκB* NFκB small interfering RNA, *HUVECs* human umbilical vein endothelial cells, *q-PCR* Quantitative real time PCR

Table S1.

Patients for single-cell RNA-seq analysis.

In this study, we integrated our original dataset with a publicly available scRNA-seq dataset (GSE156326), totaling 12 samples (6 normal skin, 6 hypertrophic scars).

In our dataset, the normal skin donors ranged in age from 37 to 48 years, with two females and one male, all of Han Chinese ethnicity. The sampling sites were the abdomen or lower limbs. In contrast, the patients with hypertrophic scars were aged between 25 and 44 years, consisting of two females and one male, all of Han Chinese ethnicity. Their scar sampling sites were all located on the limbs (two on the lower limbs and one on the forearm), and the duration of the hypertrophic scars ranged from 7 to 9 months, which aligns with the proliferative phase of hypertrophic scar formation (≤1 year).

| Tissue  (n=3 per group) | Age | Sex | Race | Site of collection | Time since injury |
| --- | --- | --- | --- | --- | --- |
| Normal skin | 48 | Female | Chinese Han population | Abdomen | — |
|  | 37 | Female | Chinese Han population | Lower limb | — |
|  | 40 | Male | Chinese Han population | Lower limb | — |
| Hypertrophic scar | 27 | Female | Chinese Han population | Lower limb | 9 months |
|  | 44 | Female | Chinese Han population | Lower limb | 7 months |
|  | 25 | Male | Chinese Han population | forearm | 9 months |

*scRNA-seq* Single-cell RNA-seq

Table S2.

Patients for Bulk RNA sequence analysis.

The normal skin donors ranged in age from 27 to 43 years, with two females and one male, all of Han Chinese ethnicity. The sampling sites were the single eyelids or lower limbs. In contrast, the patients with hypertrophic scars were aged between 28 and 35 years, consisting of two females and one male, all of Han Chinese ethnicity. Their scar sampling sites were the neck, forearm, and lower limbs (common locations for hypertrophic scars), and the duration of the scars ranged from 9 to 10 months, which aligns with the proliferative phase of hypertrophic scar formation (≤1 year).

| Tissue  (n=3 per group) | Age | Sex | Race | Site of collection | Time since injury |
| --- | --- | --- | --- | --- | --- |
| Normal skin | 27 | Female | Chinese Han population | Lower limb | — |
|  | 30 | Female | Chinese Han population | Lower limb | — |
|  | 43 | Male | Chinese Han population | Single Eyelid | — |
| Hypertrophic scar | 35 | Male | Chinese Han population | Neck | 10 months |
|  | 28 | Female | Chinese Han population | forearm | 9 months |
|  | 32 | Female | Chinese Han population | Lower limb | 9 months |

Table S3.

Characteristics of 20 hypertrophic scar patients.

The 20 donors with hypertrophic scars ranged in ages from 5 to 47 years, comprising 10 females and 10 males. All of them were of Han Chinese ethnicity. The scar sites included five on the neck, four on the face, seven on the abdomen, and four on the anterior chest (common locations for hypertrophic scars). The duration of the scars ranged from 6 to 12 months, which aligns with the proliferative phase of hypertrophic scar formation (≤1 year).

A multifaceted approach is typically employed to address the criteria for diagnosing and classifying hypertrophic scars in human subjects, combining clinical evaluation, standardized scoring systems, and histopathological analysis. Clinically, hypertrophic scars are identified by their elevated, erythematous, and firm appearance, confined to the original wound boundaries, often accompanied by symptoms such as pruritus or pain. To ensure accuracy, a skin biopsy may be performed to differentiate hypertrophic scars from other conditions, such as keloids (which extend beyond wound margins). Quantitative assessment is further supported by established scoring systems, such as the VSS or the POSAS, which provide subjective and objective evaluations for clinical and research purposes. Histopathologically, hypertrophic scars are characterized by thickened dermis with collagen nodules, myofibroblasts, and increased vascularity, distinguishing them from non-hypertrophic scars. For inclusion in studies, scars must meet specific criteria, such as being active (elevated, erythematous, and symptomatic), and differential diagnosis is critical to exclude other entities, such as keloids, dermatofibromas, or sarcomas, based on clinical and histological features. This integrated framework ensures a structured and reliable classification of hypertrophic scars.

| Patients  (n=10) | Age | Sex | Race | Pathogenic site | Time since injury |
| --- | --- | --- | --- | --- | --- |
| #1 | 6 | Male | Chinese Han population | Neck | 6 months |
| #2 | 5 | Male | Chinese Han population | Abdomen | 9 months |
| #3 | 40 | Female | Chinese Han population | Abdomen | 11 months |
| #4 | 31 | Male | Chinese Han population | Abdomen | 7 months |
| #5 | 29 | Female | Chinese Han population | Neck | 7 months |
| #6 | 47 | Male | Chinese Han population | Face | 9 months |
| #7 | 25 | Female | Chinese Han population | Chest | 8 months |
| #8 | 20 | Female | Chinese Han population | Abdomen | 10 months |
| #9 | 25 | Female | Chinese Han population | Face | 9 months |
| #10 | 33 | Male | Chinese Han population | Neck | 7 months |
| #11 | 17 | Male | Chinese Han population | Chest | 7 months |
| #12 | 25 | Female | Chinese Han population | Neck | 11 months |
| #13 | 31 | Male | Chinese Han population | Chest | 6 months |
| #14 | 34 | Male | Chinese Han population | Face | 8 months |
| #15 | 25 | Female | Chinese Han population | Abdomen | 9 months |
| #16 | 27 | Male | Chinese Han population | Face | 9 months |
| #17 | 19 | Male | Chinese Han population | Abdomen | 8 months |
| #18 | 40 | Female | Chinese Han population | Chest | 7 months |
| #19 | 18 | Female | Chinese Han population | Abdomen | 9 months |
| #20 | 31 | Female | Chinese Han population | Neck | 12 months |

*VSS* Vancouver Scar Scale, *POSAS* Patient and Observer Scar Assessment Scale

Table S4.

Primer sequences of target genes.

| Species | Gene  name | Forward primer | Reverse primer |
| --- | --- | --- | --- |
| human | GAPDH | GGAGCGAGATCCCTCCAAAAT | GGCTGTTGTCATACTTCTCATGG |
| human | TRPV1 | TGCGGTCAAGCAGAGTTTCA | CGGCAGGACTCTTGAAGACC |
| human | IL-6 | CTCCTTCTCCACAAGCGCC | GGGCGGCTACATCTTTGGAA |
| human | CD31 | CTGAGGAAAGCCAAGGCCAA | CGTCTGAGTTCAGAGGCTCTTT |
| human | VEGFA | ACAACAAATGTGAATGCAGACCA | GAGGCTCCAGGGCATTAGAC |
| human | CD34 | TGATTGCACTGGTCACCTCG | ATAAGGGTCTTCGCCCAGCC |
| human | VEGFR | GAGGGGAACTGAAGACAGGC | GGCCAAGAGGCTTACCTAGC |
| human | eNOS | CCGGAGAATGGAGAGAGCTT | AGTGGGTCTGAGCAGGAGAT |
| rabbit | β-actin | GCAGAAACGAGACGAGATTG | GCAGAACTTTGGGGACTTTG |
| rabbit | col1a1 | CTGGTGCCAAGGGTCTCAC | ACGCCTCTCTCTCCAGCTTT |
| rabbit | TGF-β | AGTGGACATCAACGGGATCAG | AGCAGTTCTTCTCTGTGGAGC |
| rabbit | Fibronectin | GGCAATTGACTTTGAGCAGGA | GGCTAAGCTGACTCTGAACTC |
| rabbit | α-SMA | AACCCTGTTGACTGAGGCAC | AGTCCAGCACAATGCCAGTT |
| rabbit | TRPV1 | TCTGCTCAAAGCCATGCTCA | AGAGCTGTCTGGCCCTTGTA |
| rabbit | IL-6 | CCAGGCAGAACCATCGAGAG | CTCAGCAGGCAGGTCTCATTA |
| rabbit | CD31 | AGCACCACTTCTGAACTCCA | TGTTTTCCATGAAATCTCGGTGT |
| rabbit | VEGFA | CGCAAGAAATCCCGTCCCT | GCCTCGGCTTGTCACATCT |
| rabbit | CD34 | CAAGCCACCAGAGCTACTCC | ATAAGGGTCTTCGCCCAGCC |
| rabbit | VEGFR | GCCTCTGTGGGTTTGCCT | CTCTGATGGTTGGGCCAGAG |
| rabbit | eNOS | GCCAGAGCAGCACAAGAGTT | CTAGAGCCCCTGCACTGTC |

Table S5.

Primer sequences of SiRNAs target genes NF-κB.

| Species | Gene  name | Sense primer (5’-3’) | Antisense primer (5’-3’) |
| --- | --- | --- | --- |
| human | Si-NFκB #1 | /rG//rG//rG//rA//rG//rG//rA//rA//rA//rU//rU//rU//rA//rU//rC//rU//rU//rC//rU/TT | /rA//rG//rA//rA//rG//rA//rU//rA//rA//rA//rU//rU//rU//rC//rC//rU//rC//rC//rC/TT |
| human | Si-NFκB #2 | /rC//rA//rG//rU//rG//rU//rC//rU//rU//rA//rC//rA//rC//rU//rU//rA//rG//rC//rA/TT | /rU//rG//rC//rU//rA//rA//rG//rU//rG//rU//rA//rA//rG//rA//rC//rA//rC//rU//rG/TT |
| human | Si-NFκB #3 | /rC//rA//rG//rG//rU//rA//rU//rU//rU//rG//rA//rC//rA//rU//rA//rU//rU//rA//rA/TT | /rU//rU//rA//rA//rU//rA//rU//rG//rU//rC//rA//rA//rA//rU//rA//rC//rC//rU//rG/TT |

*SiRNA* small interfering RNA
